# Long-chain fatty acid- and acylcarnitine-bound oxygenated myoglobin interactions with outer mitochondrial membrane

**DOI:** 10.3389/fbinf.2026.1986755

**Published:** 2026-10-16

**Authors:** Andriy Anishkin, Sangita Kachhap, Kiran Kumar Adepu, Ryan Sturms, Vaidehi Ulaganathan, Sree V. Chintapalli

**Affiliations:** 1 Department of Biology, University of Maryland, College Park, MD, United States; 2 Duke Human Vaccine Institute, Duke University School of Medicine, Durham, NC, United States; 3 Arkansas Children’s Nutrition Center, Little Rock, AR, United States; 4 Department of Pediatrics, University of Arkansas for Medical Sciences, Little Rock, AR, United States; 5 Department of Chemistry and Physics, Drake University, Des Moines, IA, United States; 6 Department of Food Science Nutrition, Faculty of Applied Sciences, UCSI University, Kuala Lumpur, Malaysia

**Keywords:** mitochondria, molecular dynamic (MD), myoglobin, palmitate, palmitoyl carnitine

## Abstract

**Introduction:**

Oxygenated myoglobin (oxy-Mb) releases oxygen (O_2_) during interactions with the outer mitochondrial membrane (OMM) and is shown to bind hydrophobic metabolic substrates like palmitate (PLM) and palmitoylcarnitine (PLC). However, it remains unclear how the binding of these specific lipids influences oxy-Mb interaction with the OMM.

**Methods:**

We employed molecular dynamics (MD) simulations to evaluate PLM- and PLC-bound oxy-Mb/membrane interactions in two settings: an average-composition OMM and a cardiolipin-enriched contact-site OMM (CS-OMM). Following steered MD-assisted contact establishment, we assessed unrestrained membrane-association stability and steered detachment forces.

**Results:**

Both PLM- and PLC-bound oxy-Mb, unrestrained after attachment to the membrane, demonstrated a strong preference for the CS-OMM, showing 2.3- to 2.7-fold increases in stability compared to the average-OMM. However, the bound lipid influenced membrane-contact persistence and resistance to steered detachment. PLM-bound oxy-Mb was 10%–20% more stable overall and required approximately twice the detachment force of the PLC-bound complex. During steered detachment, it required roughly twice the force of the PLC-bound complex and preferentially interacted with cardiolipin. Conversely, PLC-bound oxy-Mb exhibited weaker membrane association and preferentially engaged phosphatidylethanolamine and phosphatidylinositol.

**Discussion:**

The identity of the bound lipid actively modulates oxy-Mb’s membrane stability, phospholipid preference, and membrane facing orientation. PLM promotes a more mechanically robust membrane association, whereas PLC produces a distinct and weaker interaction pattern. These structural differences may influence the accessibility of the heme region at the mitochondrial surface; however, O_2_ release and lipid transfer were not directly measured in the present simulations.

## 1 Introduction

Coordinated flow of oxygen (O_2_) and metabolites are crucial for energy production and are finely regulated depending on energy needs of a cell. The globin family proteins play a critical role in the O_2_ transport and homeostasis (Richardson et al., 1995; Yang and Phillips, 1996; Richardson et al., 1999; Merx et al., 2001; Gell, 2018), and more recently members of this family have been discovered to participate in metabolite binding/transport as well (Sriram et al., 2008; Reeder et al., 2011; Shih et al., 2014; Chintapalli et al., 2015; Shih et al., 2015; Chintapalli et al., 2016; Jue et al., 2016; Jue et al., 2017; Chintapalli et al., 2018; Adepu et al., 2022a; b). In addition to its function in storing and transporting O_2_, myoglobin (Mb), a heme-containing protein predominantly found in cardiac and skeletal muscles, also interacts with gaseous ligands like carbon monoxide (CO) and nitric oxide (NO), making it pivotal in intracellular gas transport and storage (Collman et al., 1979; Eich et al., 1996; Nakamura and Nakamura, 1996; Plotnikov et al., 2009). The heme ring resides within Mb’s structure, shielded by hydrophobic residues, safeguarding it from the aqueous environment and preventing direct access by ligands in solution (Kendrew et al., 1958; Vinogradov et al., 1993; Suzuki and Imai, 1998). Mb knockout (Mb−/−) mice, characterized by the absence of Mb expression, exhibit a unique response in terms of cardiac muscle oxidative metabolism when subjected to normoxic conditions (Merx et al., 2005; Jue et al., 2016). Remarkably, these mice seem to maintain their oxidative metabolism despite the lack of Mb, suggesting the presence of compensatory mechanisms (Garry et al., 1998; Garry et al., 2000). Notably, these compensatory adaptations include an observed increase in capillarity, which is presumed to serve the purpose of minimizing the distance for efficient O_2_ diffusion between the bloodstream and mitochondria (Grange et al., 2001; Meeson et al., 2001).

The physiological importance of maintaining Mb structural and functional integrity is further illustrated by myoglobinopathy, an autosomal-dominant muscle disease caused by the recurrent p.His98Tyr mutation in human Mb. The mutation alters O_2_ binding, accelerates heme dissociation, affects redox properties, and increases the propensity of Mb to aggregate, ultimately producing progressive skeletal-muscle disease with variable cardiac involvement (Olive et al., 2019; Hama et al., 2022; Kachhap et al., 2026). These observations emphasize that perturbations of Mb structure and its heme environment can have important physiological consequences and further support the need to understand factors, including metabolite binding and membrane interactions, that modify Mb behavior. Increasing evidence indicates that Mb expressed in cardiac and skeletal tissues, binds fatty acids (FAs) in its oxygenated state (oxy-Mb) and releases them upon conversion to deoxygenated Mb (deoxy-Mb) (Chintapalli et al., 2015; Chintapalli et al., 2018). Backing this notion, biochemical and NMR investigations have showcased the *in vitro* engagement between oxy-Mb and lipid and lipid derivatives (Sriram et al., 2008; Shih et al., 2014; 2015; Chintapalli et al., 2016; Jue et al., 2017). Recent findings using molecular dynamic (MD) simulations have predicted the interaction site where medium- and long-chain FAs bind to oxy-Mb and identified key amino acid residues in the formation of the oxy-Mb + FA complex (Chintapalli et al., 2016; Jue et al., 2017; Chintapalli et al., 2018). Besides FAs, oxy-Mb also exhibits analogous binding interactions with both medium- and long-chain acylcarnitines (ACs) (Chintapalli et al., 2016; Chintapalli et al., 2018). Subsequent findings from molecular modeling and isothermal titration calorimetric (ITC) studies substantiate the binding of both FAs and ACs to oxy-Mb, where ACs possess higher binding affinities toward oxy-Mb compared to FAs (Chintapalli et al., 2016). Considering the experimental findings concerning the O_2_ release pathway adjacent to the His-E7 gate in mammalian cells (Olson et al., 1988; Scott et al., 2001; Dantsker et al., 2005; Olson et al., 2007; Birukou et al., 2010; Birukou et al., 2011) and the co-localized binding of FAs and ACs (Chintapalli et al., 2016; Chintapalli et al., 2018), structural analysis implies that these lipids might significantly impact the O_2_ dynamics and release. This influence could arise from lipid induced alterations in O_2_ diffusion pathways, including partial obstruction or changes in local protein dynamics, which may either stabilize O_2_ binding or facilitate its release depending on the bound lipid and protein conformation.

Furthermore, recent experimental results suggest that O_2_ transport into the mitochondria involves interaction of Mb with the mitochondrial membrane (Postnikova et al., 2009; Yamada et al., 2013). During the transfer of O_2_ from Mb to mitochondria, oxy-Mb engages with negatively charged phospholipids of the outer mitochondrial membrane (OMM), involving electrostatic interactions that contribute to the conversion to deoxy-Mb (Postnikova and Tselikova, 2005; Yamada et al., 2013). Immunohistochemical and electron microscopy investigations have revealed the co-localization of Mb and mitochondria during the process of O_2_ exchange (Postnikova and Shekhovtsova, 2012; Yamada et al., 2013). Recently, we conducted MD simulations using a heterogeneous OMM and contact-site OMM (CS-OMM, due to the presence of the negatively charged lipid molecule cardiolipin, CL) to predict interactions with deoxy- and oxy-Mb (Anishkin et al., 2023). Due to the structural dissimilarity between the two membranes (representing distinct regions of the OMM), the difference in CL concentration between them raised inquiries regarding the influence of CL in molding mitochondrial membrane structure (Wilson et al., 2019; Oliveira et al., 2022). Aligned with the distinction in membrane structure, our recent *in silico* MD simulation results regarding Mb’s interaction with the OMM demonstrated that deoxy-Mb displayed a comparatively stable attachment to the OMM, whereas oxy-Mb displayed enhanced attachment at the CS-OMM (Anishkin et al., 2023). Deoxy-Mb’s attachment at average-composition sites was stabilized through interactions with phosphatidylcholine (PC) and phosphatidylethanolamine (PE) headgroups, while oxy-Mb’s binding at CS-OMM was significantly impacted by charged lipids, specifically CL (Anishkin et al., 2023).

Subsequent experimental studies have provided direct support for the functional significance of Mb-membrane association. Using (palmitoyl-oleoyl-phosphatidylcholine; POPC/palmitoyl-oleoyl-phosphatidylethanolamine; POPE) liposomes as a simplified model of the OMM, Di Lella *et al.* demonstrated that membrane association induces subtle structural perturbations within the oxy-Mb heme environment and approximately doubles the rate of O_2_ dissociation, while having little effect on O_2_ association (Di Lella et al., 2026). Resonance Raman spectroscopy, in combination with classical MD and QM/MM (Quantum Mechanics/Molecular Mechanics) simulations, suggested that these changes are primarily due to modifications of the heme environment and porphyrin geometry induced by the membrane, which leads to a reduced O_2_ affinity. Complementary surface-enhanced resonance Raman studies with membrane-mimicking interfaces also showed that the interactions between Mb and the surface cause the weakening of the Fe-O_2_interaction and enhance O_2_ release, and the MD simulations identified the lysine-rich regions in helix F and the loop connecting helices C and D (CD region) as recurrent sites of surface interaction (Zitare et al., 2026). Results support the concept that the mitochondrial membrane is not just a passive docking site for Mb but can alter the structure of the heme and therefore affect O_2_ release.

These observations suggest that oxy-Mb binding to FAs and their subsequent interaction with outer mitochondrial membrane may coordinate the simultaneous transport, but sequential release of O_2_ and FAs. Although all gas ligands ultimately bind to the heme, their diffusion through Mb’s protein matrix for entry and exit remains partially understood (Blouin et al., 2010; Birukou et al., 2011; Salter et al., 2012). The prevalent mechanism suggests ligand movement into and out of Mb is guided by the outward rotation of the His64 side chain (E7 channel), with indications of other less favorable diffusion routes within the protein network (Scott et al., 2001; Dantsker et al., 2005; Olson et al., 2007). Nonetheless, investigations into the entry and exit routes of O_2_ within globin family proteins have solely focused on scenarios involving its binding to gaseous ligands, omitting examinations of when oxy-Mb is either bound to secondary metabolites or the mitochondrial membrane. Although recent studies now demonstrate that membrane association itself can alter the heme environment and facilitate O_2_ release (Di Lella et al., 2026; Zitare et al., 2026), these investigations have largely examined Mb in the absence of a bound fatty-acid-derived metabolite. It is therefore unclear whether the binding of the FA/AC-binding regions affects the stability, lipid selectivity or orientation of oxy-Mb at the mitochondrial surface, especially in CL-enriched contact sites. This distinction may be significant as the metabolite-induced changes in Mb orientation could affect the spatial relationship between the heme crevice and the membrane, affecting the availability of pathways for the O_2_ release and at the same time positioning the bound lipid for transfer towards the mitochondrial membrane.

To address this knowledge gap, in the present study, we investigated how the binding of lipid-derived metabolites influences the interaction of oxy-Mb with the mitochondrial outer membrane. We employed molecular dynamics simulations of oxy-Mb bound to palmitate (PLM) or palmitoylcarnitine (PLC) interacting with two physiologically relevant membrane environments: an average-OMM, representing the general OMM, and a CL-enriched contact-site, CS-OMM, representing the specialized OMM region at a mitochondrial contact site. Using multiple initial orientations of each Mb-lipid complex, we evaluated the stability and strength of membrane association, identified preferential protein- and membrane-lipid interaction sites, and characterized the orientation of the Mb heme region relative to the membrane interface. By directly comparing PLM- and PLC-bound oxy-Mb, we aimed to determine whether the identity of the bound lipid modulates Mb-membrane recognition and whether such interactions could provide a structural basis for coordinating the delivery of O_2_ and lipid-derived metabolic substrates to mitochondria.

## 2 Methods

### 2.1 Construction of outer mitochondrial membrane

In the present study, mitochondrial outer membrane models were constructed and characterized as previously described (Anishkin et al., 2023). Briefly, two lipid environments were considered: an average-composition outer mitochondrial membrane (average-OMM) and a contact-site outer mitochondrial membrane (CS-OMM), representing regions of the OMM associated with mitochondrial contact sites. The lipid compositions were compiled from the existing rodent mitochondrial lipidomic and biochemical data that reveal the abundance of phospholipid headgroups, membrane asymmetry, and enrichment of specific lipid species at the mitochondrial contact regions (Stefanyk et al., 2010; Horvath and Daum, 2013; Basu Ball et al., 2018; Wilson et al., 2019; Oliveira et al., 2022; Anishkin et al., 2023). The detailed composition of lipid headgroups and acyl chains of the average- and CS-OMM models is presented in Supplementary Table S1.

The membrane patches were constructed using the CHARMM-GUI Membrane Builder and solvated in an aqueous solution containing 150 mM NaCl (Klauda et al., 2010; Pandit and Klauda, 2012; Lemmin et al., 2013; Venable et al., 2014). The average- and CS-OMM systems were equilibrated for 50 and 100 ns, respectively, until membrane properties, including area per lipid and membrane hydration, reached stable values (Supplementary Figure S1). The longer equilibration of the CS-OMM model reflected its higher CL abundance and the tendency of CL molecules to form local clusters. To increase sampling efficiency, the cytoplasm-facing lipid composition was reproduced on both sides of each membrane patch. Although the two monolayers had the same target lipid composition, lipid positions were independently randomized during membrane construction. This arrangement enabled two oxy-Mb–lipid complexes to interact simultaneously with opposite membrane surfaces while experiencing distinct local lipid environments (Anishkin et al., 2023). Both systems were equilibrated for an additional 10 ns prior to the addition of the Mb complexes before symmetric OMM and CS-OMM systems were obtained. Only membrane phospholipids were included in the present models; integral and peripheral OMM proteins were excluded to specifically examine lipid-mediated Mb-membrane interactions.

### 2.2 Construction of PLM- and PLC-bound oxy-Mb models

The starting structures of mouse oxy-Mb complexed with PLM or PLC were obtained from our previously characterized Mb-lipid models (Chintapalli et al., 2018). These structures held the bound O_2_ molecule at heme and the corresponding lipid ligand in our previously identified Mb-binding region. Each protein-ligand complex was hydrated and incorporated into corresponding OMM or CS-OMM simulation system. Specific numbers of counterions were added for each system to maintain overall system electroneutrality, whereas NaCl was included to achieve a final ionic strength of approximately 150 mM. To sample different possible modes of interaction between lipid-bound oxy-Mb and the membrane, six orthogonal starting orientations of each protein-ligand complex were generated, with different Mb surfaces facing the bilayer, similar to our previous study (Anishkin et al., 2023). For each membrane composition, six system-level simulations were performed for PLM-bound oxy-Mb and six for PLC-bound oxy-Mb. Therefore, the study comprised 24 independently propagated system-level simulations across the two membrane compositions. Each simulation contained two oxy-Mb-lipid complexes interacting with opposite membrane surfaces, yielding up to 12 Mb-membrane observations per lipid/membrane condition and 48 protein-membrane observations in total. The two observations obtained from the same simulation box were treated as paired observations rather than as separate system-level replicates. Separate simulation trajectories were initiated from each orientation for both PLM-bound and PLC-bound oxy-Mb. For all systems with 2 Mb complexes on opposite sides of the membrane, both protein molecules were initially oriented the same way with respect to the surface of the membrane. However, the lipid environment in which each protein operated varied laterally, since the lipid contents in different membranes were not uniformly distributed. Protein replicas and their periodic images were placed at a minimum distance of about 25–30 Å apart between the nearest protein surfaces, to minimize direct protein-protein interactions.

### 2.3 Molecular dynamic (MD) simulations

MD simulations were carried out with Nanoscale Molecular Dynamics, NAMD (Phillips et al., 2005; Melo et al., 2018), in the computational framework developed for interactions of Mb with OMM (Anishkin et al., 2023), with the main difference being the use of PLM- or PLC-bound oxy-Mb complexes. Molecular visualization, trajectory processing and subsequent interaction analyses were carried out in Visual Molecular Dynamics (VMD) with custom Tcl scripts (Humphrey et al., 1996). All simulations were performed using CHARMM36 force-field parameters under periodic boundary conditions with the TIP3P water model (Huang et al., 2017). Parameters for the O_2_-bound heme complex were retained from the previously validated oxy-Mb model (Daigle et al., 2009). The systems were simulated under isothermal-isobaric conditions, with pressure maintained at 1 atm and temperature maintained at 300 K using Langevin dynamics. Long-range electrostatic interactions were evaluated using the particle-mesh Ewald method, and short-range nonbonded interactions were treated using a 12 Å cutoff with switching of the interactions near the cutoff distance. Prior to production simulations, each protein-lipid-membrane system was energy minimized to eliminate unfavorable atomic contacts. The minimization procedure followed the previously described protocol in which solvent was initially relaxed while the protein was restrained, followed by relaxation of protein hydrogens and solvent and, finally, unrestricted minimization of the complete system (Anishkin et al., 2023). Although the six initial protein orientations were predefined rather than randomly generated, substantial configurational diversification occurred during the 40-ns low-force membrane-approach phase. Thermal motion produced protein rotational changes of up to approximately 90°, lateral protein displacement on the order of 1 nm, and lateral lipid diffusion of approximately 1–3 nm, as illustrated in Supplementary Figure S2. In an additional validation set of five simulations initiated from identical starting coordinates and subjected to the same approach protocol, the resulting membrane-bound configurations showed negligible overlap in protein-lipid contact pairs, demonstrating substantial divergence of initially identical systems during the contact-establishment phase.

At the beginning of each protein-membrane association simulation, the center of mass for PLM- or PLC-bound oxy-Mb complex was positioned approximately 3 nm from the membrane surface, maintaining approximately 4–6 intervening layers of water between the closest protein and lipid atoms. The simulation protocol included three steps: (i) membrane approach and contact establishment, (ii) unrestrained relaxation of the resulting Mb-membrane complex, and (iii) steered-detachment of Mb from the membrane. The first stage involved the progressive migration of each Mb complex toward the membrane applied over a 40-ns period with a weak constant force of 0.01 kcal/mol/Å applied uniformly over the protein backbone. The total force was estimated to be about 11.33 pN per protein. The side chains, heme prosthetic group, bound O_2_ molecule, and the PLM or PLC ligand were not directly pulled by the steering force and were therefore allowed to move freely during approach and contact formation. This weak-force approach allows substantial translational and rotational adjustment of Mb as well as lateral rearrangement of membrane lipids during the 40-ns contact-establishment period (Supplementary Figure S2), thereby reducing the dependence of the final membrane-contact configuration on the initial protein orientation. The external force was then withdrawn, and each system was simulated for a further 40 ns without restraints to confirm membrane contact stabilization and test interaction persistence. Subsequently, a 30-ns steered MD simulation was performed to separate the protein complex from the membrane and quantify the relative strength of membrane association.

### 2.4 Protein-membrane contact analysis

Protein-membrane contacts were analyzed using the general contact-analysis framework described previously (Anishkin et al., 2023), with separate analyses performed for PLM- and PLC-bound oxy-Mb interacting with both average- and CS-OMM. Contacts between non-hydrogen atoms of Mb and lipids in the membrane are identified using distance criteria, which corresponds to the first coordination shell of the interacting atomic groups. If atom-type specific thresholds were needed, cutoff distances were calculated by radial distribution functions and set at the first minimum after major contact peak. The trajectories were analyzed for the appearance of atomic contacts between the membrane and Mb structure, and these contacts were assigned to the corresponding Mb residue and membrane lipid. The contact frequencies were determined as the number of analyzed trajectory frames in which a particular protein residue interacts with the membrane at least once. The contact frequencies on the residues were then averaged over the independent starting orientations of distinct combinations of Mb-lipid-membrane to map the regions of membrane interaction for each residue. Finally, the contact-frequency values were mapped onto the three-dimensional structure of Mb using VMD to visualize regions preferentially involved in association with the average- or CS-OMM. The analyses were conducted separately for all PLM- and PLC-bound oxy-Mb structures to determine whether the identity of bound lipid altered the distribution of Mb-membrane contacts.

### 2.5 Stability of Mb-membrane association

The persistence of each PLM- or PLC-bound oxy-Mb interaction with the membrane was evaluated during the 40-ns unrestrained relaxation phase. For each trajectory, the total number of protein-membrane atomic contacts was monitored over time. Interactions that retained a substantial number of contacts throughout the relaxation period were considered more stable, whereas progressive loss of contacts indicated weakening of membrane association or spontaneous detachment. To compare trajectories that initially contain different numbers of membrane contacts, an attachment stability score was calculated as:

$$S_{\text{stability}}=\left(1+N_{\min}\right)/N_{\max}$$

where N_min_ and N_max_ correspond to the minimum and maximum numbers of protein-membrane contacts observed during the 40-ns unrestrained trajectory, respectively. This metric was intentionally selected to normalize contact persistence across trajectories that established different maximum numbers of membrane contacts, while remaining sensitive to partial or complete loss of membrane association during relaxation. Thus, higher values indicate that a greater fraction of the maximum established contacts was retained throughout the trajectory. Addition of “1” to the numerator avoided a zero-stability score for trajectories in which there was complete loss of membrane contact. Stability scores were calculated independently for PLM- and PLC-bound oxy-Mb interacting with the average- and CS-OMM and are reported as percentages for comparison among systems.

### 2.6 Steered molecular dynamics and membrane detachment analysis

After the 40-ns unrestrained relaxation time, protein-membrane association of the Mb complexes was examined by steered MD to estimate the relative strength of protein-membrane attachment. A harmonic restraint was applied to the center of mass of the protein and translated away from the membrane surface at a constant velocity of 0.5 Å/ns for up to 30 ns. The harmonic restraint force constant was 10 kcal/mol/Å^2^. Rotational and lateral degrees of freedom of protein were otherwise retained during separation. The force required to move the Mb complex away from membrane was recorded throughout each trajectory. The detachment force was used as a comparative measure of mechanical resistance to membrane separation among PLM- and PLC-bound oxy-Mb under the same pulling conditions. Because steered detachment is a nonequilibrium process, these force measurements were not interpreted as equilibrium binding affinities or thermodynamics binding free energies. Trajectories that lost membrane association during the preceding unrestrained relaxation phase were excluded from the primary quantitative detachment-force analysis because no established complex remained for steered separation. Two of 12 observations were excluded, both for PLC-bound oxy-Mb at the CS-OMM; all 12 observations were retained for each of the other conditions. For sensitivity analysis, the two spontaneously detached configurations were assigned to provide a value of 0 pN, a conservative failure inclusive estimate across all 12 observations. The 0-pN assignment was used only for this analysis and does not represent a measured steered-detachment force. For direct comparison with our previous oxy-Mb study (Anishkin et al., 2023), the present simulations used the same average-OMM and CS-OMM membrane models, NAMD/CHARMM36 simulation framework, pulling velocity of 0.5 Å/ns and harmonic restraint force constant of 10 kcal/mol/Å^2^, the principal difference being the presence of bound PLM or PLC to the oxy-Mb complexes.

### 2.7 Residue- and lipid-specific contributions to Mb-membrane interactions

To identify Mb regions contributing to the persistence of membrane association, residue-specific contact frequencies were calculated for stability with the corresponding protein-membrane complexes. For each Mb residue, its mean frequency of membrane contact was combined with the correlation between residue-specific contacts and attachment stability score across the corresponding set of trajectories, thus, providing a relative measure of individual residues contribution to membrane-association stability. A complementary analysis was performed for the steered-detachment trajectories. The force to pull each of the protein complexes from the membrane was compared with the percentage of residues that contacted membrane lipids to understand the contribution of each individual Mb residue to membrane attachment. This approach minimized the contribution of contacts that occurred only occasionally, but were linked to a strongly bound configuration, and further minimized the influence of highly frequent contacts that occurred in weakly held complexes. Protein-lipid interactions were also categorized by membrane lipids: PC, PE, PI, PS, PA and CL as listed in Supplementary Table S1. Lipid-specific contact frequencies and their association with attachment stability or detachment force were calculated separately for PLM- and PLC-bound oxy-Mb interacting with the average- and CS-OMM. This analysis was used to identify membrane lipid classes preferentially associated with stabilization of each Mb-lipid complex. To facilitate transparent evaluation and independent reanalysis of these interactions, the complete underlying dataset is provided as Supplementary Table S2. The dataset contains interaction metrics for individual Mb residues, amino-acid types, individual membrane lipid species, and phospholipid headgroup classes for PLM- and PLC-bound oxy-Mb at both the average OMM and CS-OMM. For features with zero average contact frequency, the importance score was recorded as zero, whereas correlation with detachment force was considered undefined and is reported as not applicable.

### 2.8 Orientation of membrane-bound oxy-Mb

The orientation of membrane-associated PLM- and PLC-bound oxy-Mb was examined using representative configurations obtained from the unrestrained relaxation and steered-detachment trajectories. In particular, the relative location of the heme containing crevice to membrane surface was considered important as this region has been shown to be a major structural path for ligand exchange in Mb. Trajectories that were associated with a membrane for a significant amount of time and/or showed relatively high resistance to steered detachment were selected as representative membrane-bound configuration. VMD was used to examine the spatial relationship between the heme crevice and membrane interface. This included evaluating the orientation of the heme-containing region, the intervening solvent layer, and the specific Mb residues contacting the membrane. PLM- and PLC-bound complexes were compared using the same criteria to evaluate whether the bound metabolite influenced the preferred orientation of Mb at both average- and CS-OMM interfaces.

### 2.9 Sampling and statistical measures

The simulations were designed as an exploratory comparison of membrane-interaction behavior across multiple starting orientations rather than as a formally powered statistical sampling of the complete membrane-bound conformational ensemble. Six independently propagated system-level simulations were performed for each lipid/membrane condition. Each system contained 2 Mb molecules interacting with opposite membrane surfaces; therefore, the upper- and lower-surface observations were considered paired within a simulation rather than independent system-level replicates. Individual trajectory values are reported to show the observed variability among membrane-interaction configurations, together with descriptive averages. Because the sampled orientations represent only a subset of the possible Mb-membrane configurations, no inferential statistical tests were used to assign population-level significance to differences among conditions.

## 3 Results

### 3.1 Lipid-bound oxy-Mb shows differential initial interaction with average-OMM and CL-enriched CS-OMM

To examine whether the identity of the bound lipid influences oxy-Mb’s interaction with the mitochondrial membrane, PLM- and PLC-bound oxy-Mb complexes were evaluated against two previously established membrane environments: the average- and CS-OMM. After equilibration, both membrane models showed heterogeneous distributions of PC, PE, PI, PS, PA, and CL, with the CS-OMM showing the expected enrichment of CL compared with the average-OMM (Supplementary Figure S1). These equilibrated membranes were used as the starting configurations for subsequent protein-membrane association simulations. The membrane compositions and equilibration behavior were consistent with our previously established OMM models (Anishkin et al., 2023). The initial interaction of the Mb-lipid complexes with the membrane were evaluated during the 40-ns membrane-approach phase. Six different initial orientations were used for each Mb-lipid complex to sample different regions of the protein surface. Contact-frequency maps generated across the association trajectories demonstrated distinct patterns for PLM- and PLC-bound oxy-Mb (Figure 1). PLM-bound oxy-Mb displayed more extensive high-frequency membrane contacts with the CS-OMM than with the average-OMM. PLC-bound oxy-Mb, on the other hand, had nearly identical initial contact distributions for the two membrane compositions. Thus, the initial recognition of the mitochondrial membrane by lipid-bound oxy-Mb was dependent on both membrane composition and the identity of the lipid bound to Mb. These observations indicate that CL enrichment exerts a stronger influence on the initial association of PLM-bound oxy-Mb than on PLC-bound oxy-Mb. Although PLM and PLC share the same C16 palmitoyl chain, the two compounds differ substantially in their polar headgroups, suggesting that the chemical nature of the Mb-bound lipid can alter the presentation and/or orientation of the protein as it approaches the membrane.

**FIGURE 1:**
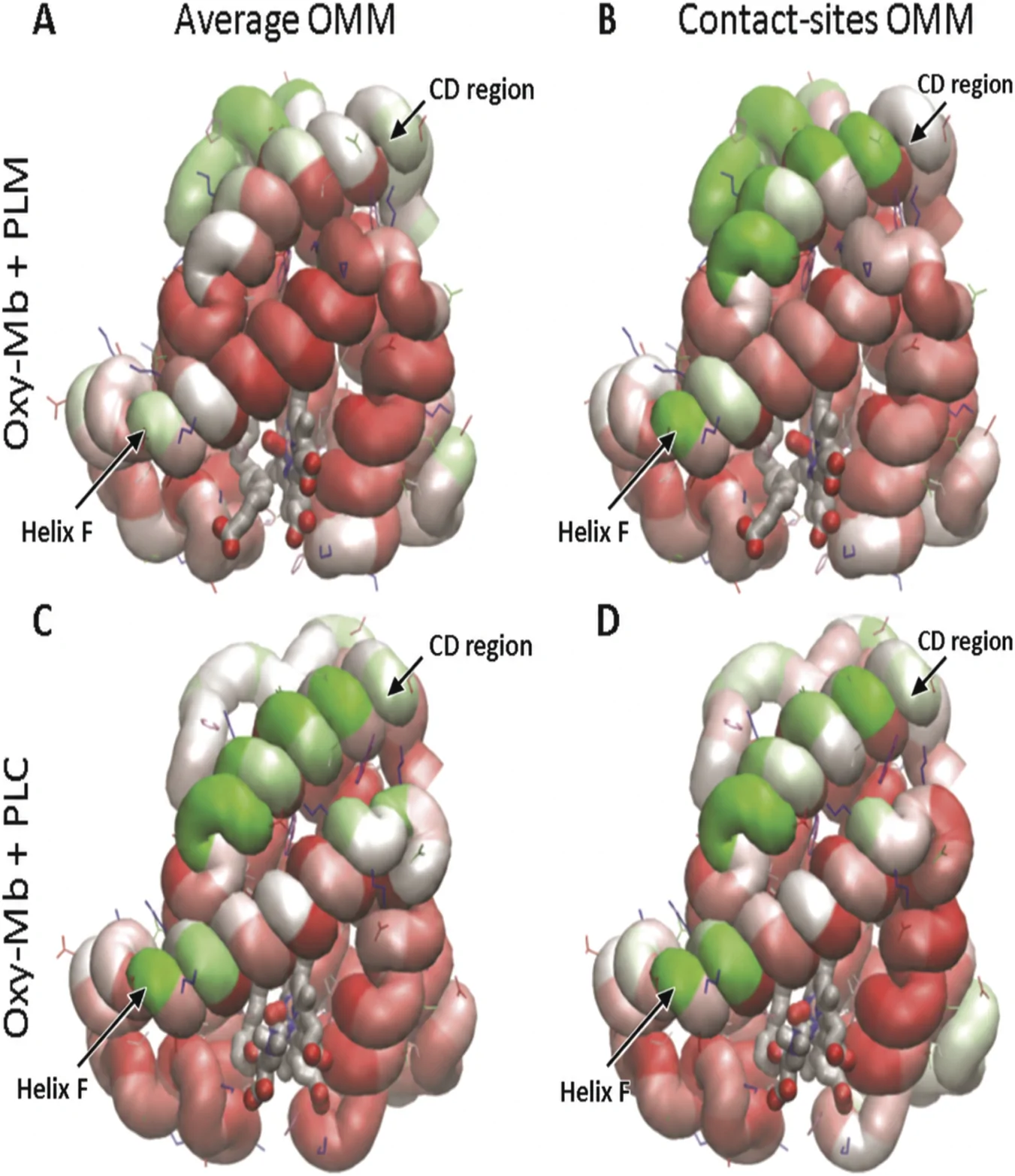
Initial Oxy-Mb-Membrane Contact Patterns. Initial contact-frequency maps of mouse oxy-Mb bound to PLM or PLC interacting with the average OMM **(A,C)** and contact-site OMM **(B,D)**. Contact frequencies between oxy-Mb residues and membrane lipids were calculated throughout the constant-force contact-establishment phase and averaged across six simulations initiated from different protein orientations for each system. Mb residues are colored according to contact frequency, ranging from low (red) to high (green), with white indicating no detectable contact contribution. The red-to-green scale is normalized to the same absolute range across all panels. Prominent membrane-contact regions include surfaces encompassing helix F and the CD region, with the extent of high-frequency contacts differing between PLM- and PLC-bound oxy-Mb.

### 3.2 PLM- and PLC-bound oxy-Mb preferentially maintain contact with CS-OMM during unrestrained relaxation

After the initial membrane-association phase, the external force was released, and each system was then allowed to undergo 40 ns of MD simulations without constraints. Two general types of behavior were observed. In stable trajectories, the number of protein-membrane contacts fluctuated around a relatively constant value and the Mb complex remained close to the membrane throughout the trajectory. In unstable trajectories, the number of atomic contacts progressively declined as the center of mass of Mb moved away from the membrane surface. In a representative unstable trajectory, the number of Mb-membrane contacts decreased from approximately 85 to 0–1 contacts, accompanied by movement of the Mb center of mass by approximately 20 Å away from the membrane. Across the independent trajectories, the minimum number of contacts retained during the 40-ns relaxation was greater at the CS-OMM for both PLM- and PLC-bound oxy-Mb (Table 1). This simpler measure of retained membrane contacts showed the same overall trend as the normalized stability score, supporting the robustness of the observed preference for the CS-OMM. Comparison of the membrane environments showed that both PLM- and PLC-bound oxy-Mb maintained more stable interactions with the CS-OMM than with the average-OMM. Based on the membrane-contact stability metric, interaction with CS-OMM was approximately 2.3-fold and 2.7-fold greater than with the corresponding average-OMM for PLM- and PLC-bound oxy-Mb, respectively (Table 2). Despite this shared preference for CS-OMM, PLM-bound oxy-Mb exhibited approximately 10%–20% greater overall membrane stability than the corresponding PLC-bound complexes. This difference was especially pronounced for oxy-Mb bound to PLC, which made initial contact with both membranes, but had a higher tendency for dissociation in the free relaxation phase. However, PLM-bound oxy-Mb showed significantly greater membrane association, particularly with CS-OMM.

**TABLE 1:** Minimum number of contacts retained by PLM- and PLC-bound oxy-Mb with the average-OMM and CS-OMM during unrestrained relaxation**.**

Independent Runs	Average OMM		Contact-sites OMM	
	Oxy-Mb + PLM	Oxy-Mb + PLC	Oxy-Mb + PLM	Oxy-Mb + PLC
	26	14	40	36
	17	14	40	22
	13	12	25	21
	9	5	23	20
	5	3	20	13
	1	0	8	6
	0	0	6	5
	0	0	4	5
	0	0	0	0
	0	0	0	0
	0	0	0	0
	0	0	0	0
Average	5.9	4.0	13.8	10.7
CS/Avg OMM Ratio			2.3	2.7
PLC/PLM Ratio		0.7		0.8

The minimum number of contacts between heavy atoms of oxy-Mb and membrane lipids was determined during the 40-ns unrestrained relaxation simulations, with contacts monitored at 5-ps intervals. Each value represents the lowest number of protein-membrane contact observed during an individual trajectory. Average values are shown for PLM- and PLC-bound oxy-Mb interacting with the average-OMM and CS-OMM. The CS/average-OMM ratio indicates the relative increase in retained contacts at the CS-OMM, while the PLC/PLM ratio compares the two lipid-bound oxy-Mb complexes within each membrane environment.

**TABLE 2:** Stability of Lipid-Bound Oxy-Mb-Membrane Interactions.

Independent Runs	Average OMM		Contact-sites OMM	
	Oxy-Mb + PLM	Oxy-Mb + PLC	Oxy-Mb + PLM	Oxy-Mb + PLC
	18.0%	11.5%	36.6%	35.6%
	15.0%	10.6%	36.6%	22.1%
	10.8%	10.2%	23.2%	21.2%
	10.6%	6.9%	22.4%	18.4%
	6.2%	5.0%	18.8%	12.7%
	2.3%	2.4%	7.9%	11.7%
	1.6%	1.7%	5.6%	7.3%
	1.3%	1.5%	4.5%	5.9%
	1.0%	1.2%	1.6%	5.6%
	1.0%	1.2%	1.4%	1.6%
	0.9%	1.0%	1.2%	1.4%
	0.8%	1.0%	1.0%	1.3%
Average	5.8%	4.5%	13.4%	12.1%
CS/Avg OMM Ratio			2.3	2.7
PLC/PLM Ratio		0.8		0.9

Stability scores of both PLM- and PLC-bound oxy-Mb interactions with the average-OMM and CS-OMM during the 40-ns unrestrained relaxation simulations following establishment of the initial membrane contact. Each value represents the stability score for an individual Mb-membrane interaction. Average stability scores are shown for each lipid-bound oxy-Mb and membrane combination. The CS/average-OMM ratio indicates the relative difference in stability between the two membrane environments, whereas the PLC/PLM ratio compares the stability of the two lipid-bound oxy-Mb complexes within each membrane composition. Cells are color-coded from lower stability (red), through intermediate stability (yellow), to higher stability (green).

### 3.3 PLM and PLC produce distinct residue- and phospholipid-specific membrane-interaction patterns

Further, to determine the regions of Mb that contribute to stabilization of the membrane-bound state, residue-level contribution maps were derived using unrestrained trajectories. For PLM-bound oxy-Mb, broader regions of positively contributing residues were observed at CS-OMM in comparison with the average-OMM. PLC-bound oxy-Mb, on the other hand, had reduced numbers of strongly stabilizing residue contributions, in accord with its enhanced ability to lose membrane association during unrestrained relaxation (Figure 2). Notably, the membrane-interacting surfaces identified in these maps included regions encompassing helix F and the CD region, with their relative contributions differing between PLM- and PLC-bound oxy-Mb. Analysis of contacts based on membrane phospholipid class also showed that membrane stabilization was different in PLM- and PLC-bound oxy-Mb. In the average-OMM, contacts were primarily from PC and PE. In contrast, at CS-OMM, oxy-Mb bound to PLM showed a significant stabilizing effect of charged phospholipids, especially CL, in addition to PC. In comparison, oxy-Mb bound to PLC had larger contributions from PE and PI, and a smaller contribution from CL. The residue- and lipid-type analysis further showed distinct patterns of contact frequency, correlation with detachment force, and relative importance between PLM- and PLC-bound oxy-Mb at the two membrane compositions (Supplementary Table S3). Among the residue types, Lys showed prominent contributions to membrane interactions, whereas the phospholipid analysis emphasized differences in PC, PE, PI, and CL contributions between the PLM- and PLC-bound complexes. These results demonstrate that binding of PLM or PLC does not simply change the magnitude of oxy-Mb membrane interaction. Instead, the two lipid structures produce distinct protein-phospholipid interaction networks, particularly at the CL-enriched CS-OMM. The complete residue- and lipid-resolved interaction dataset is provided in Supplementary Table S2. Examination of these data indicates that membrane stabilization is distributed across multiple Mb residues and lipid species rather than being attributable to a single interaction site. This distributed pattern is consistent with an orientation-dependent, multicomponent mode of membrane association in which the relative contributions of individual protein regions and phospholipid classes vary with the bound metabolite and membrane composition.

**FIGURE 2:**
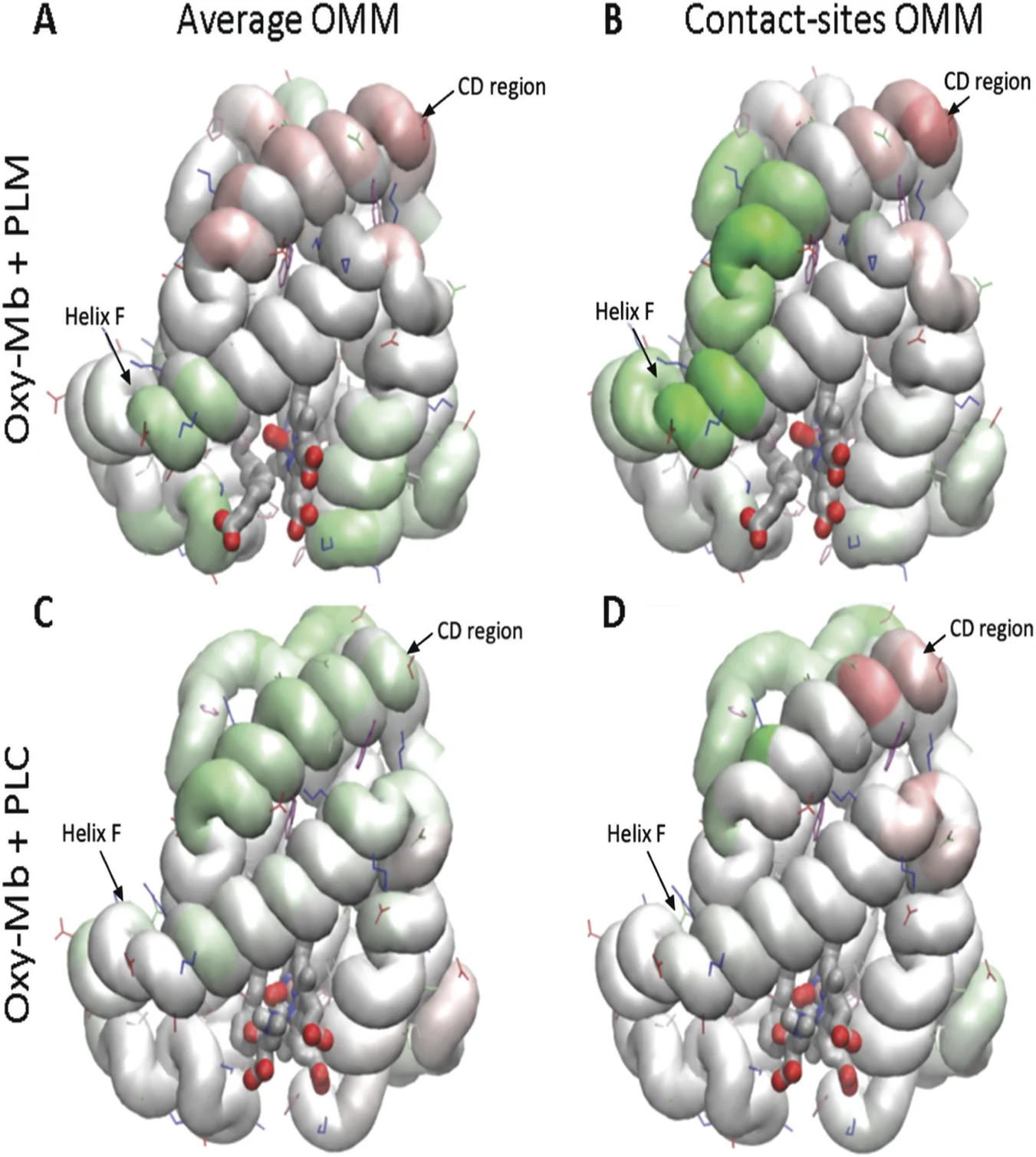
Stable Oxy-Mb-Membrane Contact Patterns. Residue-level maps of stable interactions between mouse oxy-Mb and the average-OMM or CS-OMM during the 40-ns unrestrained relaxation simulations. Mb residues are color-coded according to their contribution to membrane-association stability. Red/pink indicates residues associated with destabilization of the contact, white indicates little or no contribution, and green identifies residues contributing most strongly to stabilization of the Mb-membrane interaction. Stabilizing contacts include regions encompassing helix F and the CD region, with broader stabilizing contributions observed for PLM-bound oxy-Mb at the CS-OMM.

### 3.4 PLM markedly increases the mechanical resistance of oxy-Mb association to detachment from CS-OMM

To compare the mechanical resistance of the membrane associated complexes to separation, membrane-bound configurations that persisted through the unrestrained relaxation phase were subjected to 30 ns steered MD simulations in which the protein was systematically pulled away from the membrane. The average force required for detachment was used as a comparative measure of mechanical resistance to the membrane separation under the applied pulling protocol (Table 3). The average detachment forces were 55.5 and 40.8 pN for PLM- and PLC-bound oxy-Mb at the average-OMM, respectively, and 74.7 and 40.8 pN at the CS-OMM. For PLC-bound oxy-Mb at the CS-OMM, 10 of 12 observations (83.3%) retained sufficient membrane association for steered-detachment analysis, whereas two observations (16.7%) had already dissociated during the unrestrained relaxation phase. No observations were excluded from the other conditions. In a conservative sensitivity analysis assigning these two spontaneous-detachment events a value of 0 pN, the PLC/CS-OMM mean decreased from 40.8 to 34.0 pN. Thus, inclusion of the failed associations did not alter the direction of the PLM-PLC difference in detachment resistance. For comparison, our previously reported simulations of oxy-Mb without bound PLM or PLC, performed using the same membrane models, force field, pulling velocity, and harmonic restraint parameters, required average detachment forces of 32.4 pN at the average-OMM and 66.7 pN at the CS-OMM (Supplementary Table S4 (Anishkin et al., 2023)). Relative to this oxy-Mb reference, PLM binding was associated with greater detachment resistance at both membrane compositions, whereas PLC binding produced a smaller increase at the average-OMM but reduced the strong CS-OMM preference observed for oxy-Mb alone. PLM-bound oxy-Mb required approximately 1.3-fold greater force to detach from CS-OMM than from the average-OMM. However, the detachment forces of PLC-bound oxy-Mb were approximately equal at both compositions. Additionally, direct comparison of the two lipid-bound states showed that membrane separation for PLM-bound oxy-Mb was about twice as large as that of PLC-bound oxy-Mb for all membrane environments studied. The steered-detachment results were therefore consistent with the unrestrained relaxation simulations. PLM-bound oxy-Mb showed greater resistance to steered detachment, with the highest average detachment force observed at the CL-enriched CS-OMM. PLC-bound oxy-Mb, by comparison, showed lower detachment resistance, which was relatively insensitive to the difference between average- and CS-OMM.

**TABLE 3:** Steered-Detachment Forces of Lipid-Bound Oxy-Mb.

Independent Runs	Average OMM		Contact-sites OMM	
	Oxy-Mb + PLM	Oxy-Mb + PLC	Oxy-Mb + PLM	Oxy-Mb + PLC
	146.08	64.33	242.35	80.66
	123.29	63.43	103.38	75.27
	100.50	60.83	85.98	47.61
	45.97	58.45	82.15	42.82
	45.30	46.76	73.72	35.53
	41.05	36.39	62.19	29.49
	35.78	34.67	58.98	29.42
	34.08	34.38	54.08	28.57
	33.91	29.70	47.90	20.39
	25.07	24.81	35.92	18.00
	21.99	20.13	32.19	
	13.01	16.02	17.24	
Average	55.5	40.8	74.7	40.8
CS/Avg OMM Ratio			1.3	1.0
PLC/PLM Ratio		0.7		0.5

Average force required to detach PLM- and PLC-bound oxy-Mb from the average-OMM and CS-OMM during 30-ns steered MD simulations. Each value represents an individual detachment trajectory. Average detachment forces are shown for each lipid-bound oxy-Mb and membrane combination. Cells are color-coded from lower (red) to higher (green) detachment force. Empty cells indicate trajectories that did not retain sufficient membrane contact for quantitative analysis (NA*). Two PLC-bound oxy-Mb observations at the CS-OMM lost membrane association during the preceding unrestrained relaxation phase and therefore did not have a measurable steered-detachment force. These observations were excluded from the primary mean but were assigned 0 pN in a conservative sensitivity analysis, yielding a failure-inclusive mean of 34.0 pN.

### 3.5 PLM- and PLC-bound oxy-Mb adopt different membrane-facing orientations

The unrestrained and steered MD simulations demonstrated that the identity of the bound lipid influences both the persistence and mechanical strength of oxy-Mb association with the membrane. We next examined whether PLM and PLC also produced differences in the protein surfaces involved in membrane interaction. The residue-level interaction maps showed distinct membrane-contact patterns for PLM- and PLC-bound oxy-Mb (Figure 2), indicating that the two complexes can engage the membrane through different regions of the protein surface. PLM-bound oxy-Mb generally formed stronger and more persistent membrane interactions, whereas several PLC-bound configurations positioned different regions of the protein, including regions near the heme crevice, toward the membrane. Because O_2_ dissociation was not simulated or directly measured, these orientation differences should not be interpreted as evidence for increased or decreased O_2_ release, but rather as structural features that may influence accessibility of the heme region or ligand exchange pathways.

## 4 Discussion

In the present study, we investigated whether lipid occupancy changes the association of oxy-Mb with the OMM/CS-OMM, extending our previous analysis of oxy- and deoxy-Mb interactions with the mitochondrial membrane (Anishkin et al., 2023). The simulations suggest that both PLM- and PLC-bound oxy-Mb showed stronger membrane-contact persistence at the CS-OMM than at the average-OMM, although the nature and magnitude of these interactions differed between the two lipid-bound complexes. PLM-bound oxy-Mb showed greater contact persistence and resistance to steered detachment, particularly at the CS-OMM, whereas PLC-bound oxy-Mb showed lower detachment resistance. Lipid specific contact analysis also demonstrated that there is a larger contribution from CL to the stabilization of PLM bound oxy-Mb at the CS-OMM, whereas the contact interaction of PLC bound oxy-Mb with PE and PI was more prominent. The results indicate that the metabolite bound to oxy-Mb may affect the intensity of membrane interaction and the phospholipid dependence of this interaction. These differences in detachment force reflect relative mechanical resistance under the applied nonequilibrium pulling conditions and should not be interpreted as differences in equilibrium binding affinity or thermodynamic binding free energy. However, an important limitation of the present study is that the membrane models contain only phospholipids and exclude integral and peripheral OMM proteins. This simplified design was used intentionally to isolate lipid-mediated contributions to Mb-membrane association and the effects of membrane phospholipid composition. In intact mitochondria, membrane proteins and the more complex organization of the OMM may further influence Mb accessibility, orientation, and membrane interactions. Therefore, the differences observed here should be interpreted as properties of the simplified phospholipid membrane models and should not be directly extrapolated quantitatively to Mb interactions with intact mitochondria.

These observations agree with our previous computational study in which oxy-Mb showed a preference for the CS-OMM over the average-OMM when performing steered-detachment simulations. In that study, oxy-Mb association with CS-OMM was found to be significantly stabilized by charged phospholipids, especially CL, while larger contributions by PC and PE were observed for the average-OMM (Anishkin et al., 2023). The present results therefore indicate that binding of PLM or PLC does not remove the membrane preference which was previously observed for oxy-Mb but rather alters the interaction pattern observed. In particular, the strong contribution of CL to PLM-bound oxy-Mb indicates that the lipid metabolite carried by Mb can affect how the protein responds to differences in mitochondrial membrane composition. The difference between PLM and PLC bound oxy-Mb is striking, since both metabolites carry the same acyl chain (C16) but have significantly different polar headgroups. Previous molecular modeling and ITC experiments showed that long-chain fatty acids and acylcarnitines can bind to oxy-Mb, and that the binding affinities of the acylcarnitines tend to be higher than the corresponding free fatty acids (Chintapalli et al., 2015; Chintapalli et al., 2016; Jue et al., 2017; Chintapalli et al., 2018). Simulations also revealed that PLM and PLC are in a hydrophobic pocket of Mb near the heme and bind in similar but not identical conformations inside the protein (Chintapalli et al., 2016; Chintapalli et al., 2018). Therefore, increased membrane association observed for PLM-bound oxy-Mb is not in conflict with the increased binding of PLC to Mb. These are independent interactions, where ligand binding dictates the affinity of Mb for PLM or PLC, while the present simulations determine the affinity of the resulting Mb-ligand complex to the membrane. The additional carnitine headgroup of PLC may therefore alter the orientation or surface interaction pattern of the complex without necessarily weakening PLC binding within Mb itself. The present residue- and lipid-specific analyses further indicate that the difference between PLM- and PLC-bound oxy-Mb cannot be readily attributed to a single physicochemical interaction. Multiple protein regions and membrane lipid classes contribute to the observed membrane-associated states, suggesting combined contributions from local charged, polar, and hydrophobic interactions. However, the present sampling was not designed to quantitatively separate electrostatic, hydrophobic, and conformational energetic contributions. Such decomposition would require substantially broader sampling of membrane-bound configurations together with dedicated electrostatic, solvent-accessibility, and free-energy analyses.

The preferential interaction with the CS-OMM also appears reasonable in the context of the distinct physicochemical properties of CL-rich mitochondrial membranes. CL is an anionic phospholipid that has an impact on the organization of membranes and on the interaction with various mitochondrial proteins, and variations in the abundance or composition of CL could lead to changes in mitochondrial membrane structure and function (Chicco and Sparagna, 2007; Pennington et al., 2019). Likewise, it has been demonstrated that mitochondrial PE affects activity of the respiratory enzymes and oxidative capacity, suggesting that membrane phospholipid composition can have a direct effect on the behavior of proteins other than just as a structural background (Heden et al., 2019). Although these studies largely concern proteins of the inner mitochondrial membrane, they support the more general interpretation that different phospholipid environments can alter protein-membrane interactions. In the present simulations, this effect is reflected by the different contributions of CL, PC, PE, and PI to the PLM- and PLC-bound complexes.

The membrane orientation of the Mb complexes may also be relevant when considering O_2_ release. Overall, PLM-bound oxy-Mb associated more strongly with the membrane, while selected PLC-bound configurations had the heme crevice more oriented towards the membrane. Importantly, the present orientation analysis is qualitative and does not establish a direct relationship between heme orientation and O_2_ dissociation kinetics. The observed membrane-facing configurations should therefore be interpreted as structural differences between PLM- and PLC-bound oxy-Mb rather than as evidence for enhanced or reduced O_2_ release. Moreover, recent experimental work showed that the interaction of membrane would also influence the Mb heme environment. Di Lella *et al.* reported that interaction of oxy-Mb with POPC/POPE liposomes produced subtle structural perturbations around the heme and approximately doubled the O_2_ dissociation rate constant. They also indicated that the response is due to membrane-induced proximal effects and heme-plane distortion, based on their computational analysis (Di Lella et al., 2026). Similarly, Zitare *et al.* showed using membrane-mimicking surfaces that Mb-surface interactions can perturb the heme pocket and weaken the Fe-O_2_ interaction (Zitare et al., 2026). These studies support the possibility that the orientation and residence of PLM- and PLC-bound oxy-Mb at the membrane could be functionally relevant, but direct measurements will be necessary to determine whether the two complexes differ in their O_2_ release kinetics.

Long-chain acylcarnitines can accumulate when mitochondrial fatty acid oxidation is impaired or incompletely matched to substrate supply, including during myocardial ischemia (Fujisawa et al., 1992; Martin et al., 2000; Liepinsh et al., 2016). This provides an important physiological context for the present finding that PLM- and PLC-bound oxy-Mb do not interact equivalently with mitochondrial membranes. PLM-bound oxy-Mb formed a more persistent and mechanically resistant membrane-associated state with greater cardiolipin contribution, whereas PLC-bound oxy-Mb showed weaker association and a different phospholipid-interaction pattern. These differences suggest that changes in the relative availability of long-chain fatty acids and acylcarnitines could influence how lipid-bound oxy-Mb interacts with the mitochondrial surface. However, the present simulations do not directly measure O_2_ release, PLM/PLC transfer, or mitochondrial function, and these physiological consequences remain to be experimentally determined.

At a broader regulatory level, nutrient availability is integrated through nutrient-sensing pathways such as mechanistic target of rapamycin complex 1 (mTORC1), which responds to amino acid and metabolic signals and coordinates processes including protein synthesis, autophagy, lipid metabolism, and mitochondrial function (Fan et al., 2022; Fan and Xu, 2026; Fan et al., 2026). Recent studies illustrate how amino acid and mTORC1 signaling can translate nutrient availability into cellular metabolic responses, while dietary-restriction studies place mTOR within a broader nutrient-sensing network that regulates mitochondrial and metabolic adaptation. Although mTOR signaling was not examined in the present study, such upstream nutrient-sensing pathways may influence fatty acid utilization and the balance of lipid metabolic intermediates, thereby providing a broader regulatory context for the Mb-lipid-membrane interactions characterized in this study.

The current findings, along with previous reports, indicate that metabolite occupancy is yet another factor that affects oxy-Mb behavior at the mitochondrial surface. Both PLM and PLC share the same C16 acyl chain, but the two Mb-lipid complexes adopted different membrane-bound states, with PLM having more persistent and mechanically resistant interactions, and PLC having different phospholipid-contact and membrane-facing states. This extends our previous observation that Mb oxygenation state influences OMM recognition by showing that, within the oxygenated state, the bound metabolite can further modify membrane interactions. This could be especially important for nutrition and exercise, where modifications in fat availability in the diet or fasting/feeding state, and/or exercise intensity or duration will affect skeletal-muscle fatty-acid utilization, β-oxidation of fatty acids within the mitochondria, and the balance between free fatty acids and acylcarnitine intermediates. Increased use of fatty-acid oxidation during endurance exercise can alter the relative availability of long-chain fatty acids and acylcarnitines, which can affect the relative distribution of the oxy-Mb species at the mitochondrial surface. In contrast, when the supply of fatty acids is greater than the capacity of mitochondria to oxidize them, formation of acylcarnitine intermediates may promote a different mode of interaction between Mb and membranes. Together with recent experimental data indicating that membrane association can affect Mb O_2_ affinity (Di Lella et al., 2026; Zitare et al., 2026), the results indicate that the O_2_ delivery via Mb and the presence of lipid substrates may be coupled differently depending on fatty-acid and acylcarnitine availability. These functional consequences of the release of O_2_ or transfer of PLM/PLC have not been directly measured in the present simulations. The overall sequence of lipid-dependent membrane recognition, phospholipid-specific interaction, membrane persistence, and its proposed relationship to heme accessibility is summarized schematically in Figure 3.

**FIGURE 3:**
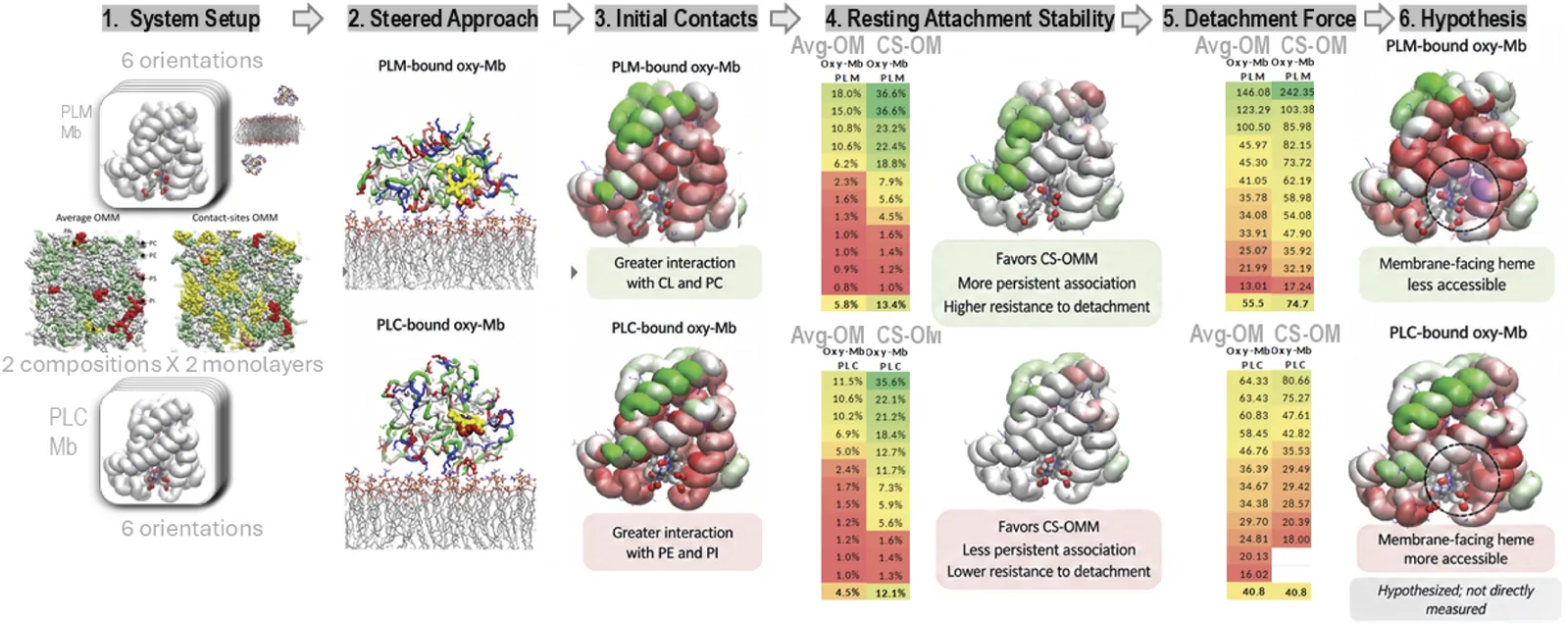
Schematic overview of the simulation workflow and proposed model for PLM- and PLC-bound oxy-Mb interactions with the mitochondrial outer membrane. (1) PLM- and PLC-bound oxy-Mb complexes were initiated from six orthogonal orientations and evaluated against average-composition OMM and cardiolipin-enriched contact-site OMM (CS-OMM) models. (2) A soft steered-approach phase allowed the complexes to establish membrane contact while retaining rotational and lateral freedom. (3) Initial contact analysis revealed lipid-dependent interaction patterns, with PLM-bound oxy-Mb showing greater contributions from cardiolipin (CL) and phosphatidylcholine (PC), whereas PLC-bound oxy-Mb showed greater contributions from phosphatidylethanolamine (PE) and phosphatidylinositol (PI). (4) During the subsequent unrestrained relaxation phase, PLM-bound oxy-Mb showed greater membrane-contact persistence, particularly at the CS-OMM, whereas PLC-bound oxy-Mb displayed lower stability and more frequent loss of membrane association. (5) Steered-detachment simulations further showed that PLM-bound oxy-Mb had greater mechanical resistance to membrane separation than PLC-bound oxy-Mb, with the highest average detachment force observed at the CS-OMM. Detachment force is interpreted as a comparative measure of mechanical resistance under the applied pulling protocol and not as an equilibrium binding affinity or thermodynamic free-energy difference. (6) Representative membrane-bound configurations suggest that PLM- and PLC-bound oxy-Mb can adopt different membrane-facing orientations, including differences in the spatial relationship between the heme-containing region and the membrane. These structural observations provide a hypothesis for potential effects on heme accessibility and O_2_ release; however, O_2_ dissociation was not directly simulated or measured in the present study.

## 5 Conclusion

This study demonstrates that the lipid metabolite carried by oxy-Mb influences its interaction with mitochondrial membranes. Although both PLM- and PLC-bound oxy-Mb preferentially associated with the CS-OMM, PLM promoted a stronger and more persistent membrane-bound state with greater CL involvement, whereas PLC produced a distinct and comparatively weaker membrane-interaction pattern. These results expand our understanding of Mb’s role beyond simple O_2_ transport, suggesting that its bound metabolic cargo actively dictates mitochondrial membrane recognition. The differential behavior of PLM- and PLC-bound oxy-Mb provides a molecular basis for future studies examining how changes in fatty acid and acylcarnitine availability influence the coordination of O_2_ and lipid-derived substrate delivery to mitochondria.

## Data Availability

The molecular dynamics simulation datasets generated and analyzed in this study, including PDB and PSF files and compressed DCD trajectories for PLM- and PLC-bound oxy-Mb interacting with average OMM and CS-OMM membrane models, are available through Zenodo at DOI: 10.5281/zenodo.22241593. The README file provided with the repository describes the dataset organization, simulation stages, and file-naming conventions.
