# Supplementary material for: Long-chain fatty acid- and acylcarnitine-bound oxygenated myoglobin interactions with outer mitochondrial membrane
Source: Front Bioinform. 2026 Oct 16;6:1986755. doi: 10.3389/fbinf.2026.1986755 (PMC13719540; doi:10.3389/fbinf.2026.1986755)
Supplement: Supplementary file 1 [file Table1.docx]

**Supplementary Table 1. Lipid Composition of Average OMM and CS-OMM.** Lipid composition of membrane patches representing the average rodent outer mitochondrial membrane (OMM) and cardiolipin-enriched contact-site OMM (CS-OMM). Cyt and MIMS denote the cytoplasmic- and mitochondrial intermembrane-space-facing leaflets, respectively; CS-Cyt and CS-MIMS denote the corresponding contact-site leaflets. Values indicate the number of each lipid species, with percentages representing the total fraction of each phospholipid headgroup within the respective leaflet. Cardiolipin enrichment in the CS-Cyt leaflet is highlighted in yellow. *Abbreviations:* PE, phosphatidylethanolamine; PC, phosphatidylcholine; PI, phosphatidylinositol; PS, phosphatidylserine; PA, phosphatidic acid; CL, cardiolipin; SAPE, stearoyl-arachidonoyl-PE; SLPE, stearoyl-linoleoyl-PE; POPE, palmitoyl-oleoyl-PE; PLPE, palmitoyl-linoleoyl-PE; SAPC, stearoyl-arachidonoyl-PC; PLPC, palmitoyl-linoleoyl-PC; SLPC, stearoyl-linoleoyl-PC; POPC, palmitoyl-oleoyl-PC; SOPI, stearoyl-oleoyl-PI; SLPI, stearoyl-linoleoyl-PI; SEPI, stearoyl-eicosatrienoyl-PI; SAPI, stearoyl-arachidonoyl-PI; POPI, palmitoyl-oleoyl-PI; SAPS, stearoyl-arachidonoyl-PS; SLPS, stearoyl-linoleoyl-PS; SLPA, stearoyl-linoleoyl-PA; DLCL, eicosadienoyl-linoleoyl-CL; POCL, palmitoyl-oleoyl-CL; SOCL, stearoyl-oleoyl-CL; SLCL, stearoyl-linoleoyl-CL.
