# Supplementary material for: Long-chain fatty acid- and acylcarnitine-bound oxygenated myoglobin interactions with outer mitochondrial membrane
Source: Front Bioinform. 2026 Oct 16;6:1986755. doi: 10.3389/fbinf.2026.1986755 (PMC13719540; doi:10.3389/fbinf.2026.1986755)
Supplement: Supplementary file 2 [file Table2.docx]

**Supplementary Table 2. Extended residue- and lipid-specific interaction dataset for PLM- and PLC-bound oxy-Mb.** Table contains interaction metrics for all 153 individual Mb residues, amino-acid types, individual membrane lipid species, and phospholipid headgroup classes for PLM- and PLC-bound oxy-Mb interacting with the average OMM and CS-OMM. Reported metrics include average contact frequency, correlation with steered-detachment force, and the corresponding importance score. Correlations are designated as not applicable when the corresponding contact frequency is zero because a meaningful correlation cannot be calculated in the absence of membrane contacts.

| **Mem**  **brane** | **Feature level** | **Feature** | **Bound lipid** | **Importance score** | **Correlation with detachment force** | **Average contact frequency** |
| --- | --- | --- | --- | --- | --- | --- |
| Average OMM | Residue | GLY1 | PLM | 0.046786076 | 0.657929187 | 0.071111111 |
| Average OMM | Residue | GLY1 | PLC | -6.19E-05 | -0.111362995 | 0.000555556 |
| CS-OMM | Residue | GLY1 | PLM | 0.096496274 | 0.7391203952990121 | 0.13055555555555542 |
| CS-OMM | Residue | GLY1 | PLC | 0.037126892 | 0.6488194733117563 | 0.057222222 |
| Average OMM | Residue | LEU2 | PLM | 0.041953063801810485 | 0.6624167968706918 | 0.063333333 |
| Average OMM | Residue | LEU2 | PLC | 0 |  | 0 |
| CS-OMM | Residue | LEU2 | PLM | 0.096703522 | 0.719282396 | 0.1344444444444443 |
| CS-OMM | Residue | LEU2 | PLC | 0.036045526 | 0.6488194733117564 | 0.055555556 |
| Average OMM | Residue | SER3 | PLM | 0.04861258 | 0.5912340786694663 | 0.082222222 |
| Average OMM | Residue | SER3 | PLC | 0.000192354 | 0.11541247704482002 | 0.001666667 |
| CS-OMM | Residue | SER3 | PLM | 0.098344697 | 0.6705320234393146 | 0.1466666666666666 |
| CS-OMM | Residue | SER3 | PLC | 0.054215267204821754 | 0.6462747084018492 | 0.083888889 |
| Average OMM | Residue | ASP4 | PLM | 0.000279902 | 0.002998955 | 0.093333333 |
| Average OMM | Residue | ASP4 | PLC | 0.021000895 | 0.5250223754745544 | 0.04 |
| CS-OMM | Residue | ASP4 | PLM | 0.10355433694115337 | 0.5371694711644848 | 0.19277777777777763 |
| CS-OMM | Residue | ASP4 | PLC | 0.066278279 | 0.4696885928239412 | 0.14111111111111088 |
| Average OMM | Residue | GLY5 | PLM | -0.011854582 | -0.29230476 | 0.040555556 |
| Average OMM | Residue | GLY5 | PLC | 0.010874153415559648 | 0.3837936499609295 | 0.028333333333333276 |
| CS-OMM | Residue | GLY5 | PLM | 0.023993303 | 0.49077210346142275 | 0.048888889 |
| CS-OMM | Residue | GLY5 | PLC | 0.047328463 | 0.604193149 | 0.078333333 |
| Average OMM | Residue | GLU6 | PLM | 0.045404995 | 0.6053999361047885 | 0.075 |
| Average OMM | Residue | GLU6 | PLC | 0.002614889 | 0.39223331512793524 | 0.006666667 |
| CS-OMM | Residue | GLU6 | PLM | 0.009775067 | 0.21993901141674374 | 0.044444444444444446 |
| CS-OMM | Residue | GLU6 | PLC | 0.047794276 | 0.6517401229118811 | 0.073333333 |
| Average OMM | Residue | TRP7 | PLM | -0.002530481 | -0.175187139 | 0.014444444444444442 |
| Average OMM | Residue | TRP7 | PLC | 0.000402169 | 0.3619523491310563 | 0.001111111 |
| CS-OMM | Residue | TRP7 | PLM | 0.087940985 | 0.8892908555254692 | 0.098888889 |
| CS-OMM | Residue | TRP7 | PLC | 0.003988076 | -0.326297131 | 0.012222222222222218 |
| Average OMM | Residue | GLN8 | PLM | -0.016069282 | -0.272874607 | 0.058888889 |
| Average OMM | Residue | GLN8 | PLC | 0.060622179 | 0.6950313541318917 | 0.087222222 |
| CS-OMM | Residue | GLN8 | PLM | 0.086232646 | 0.6929409039966119 | 0.12444444444444432 |
| CS-OMM | Residue | GLN8 | PLC | 0.055181108 | -0.415589936 | 0.1327777777777777 |
| Average OMM | Residue | LEU9 | PLM | -0.006394516 | -0.295131489 | 0.021666666666666612 |
| Average OMM | Residue | LEU9 | PLC | 0.010765566452334945 | 0.4212612959609331 | 0.025555555555555526 |
| CS-OMM | Residue | LEU9 | PLM | 0.000517861 | -0.310716551 | 0.001666667 |
| CS-OMM | Residue | LEU9 | PLC | 0.001021078 | 0.096733746 | 0.010555556 |
| Average OMM | Residue | VAL10 | PLM | 0 |  | 0 |
| Average OMM | Residue | VAL10 | PLC | 0 |  | 0 |
| CS-OMM | Residue | VAL10 | PLM | 0 |  | 0 |
| CS-OMM | Residue | VAL10 | PLC | 0 |  | 0 |
| Average OMM | Residue | LEU11 | PLM | -0.004130553 | -0.185874888 | 0.022222222222222133 |
| Average OMM | Residue | LEU11 | PLC | 0.021261365 | 0.6714115114528298 | 0.031666666666666635 |
| CS-OMM | Residue | LEU11 | PLM | 0.060616392 | 0.7273967074869364 | 0.083333333 |
| CS-OMM | Residue | LEU11 | PLC | 0.01950257 | -0.444362352 | 0.043888888888888804 |
| Average OMM | Residue | ASN12 | PLM | -0.012541942 | -0.297045995 | 0.042222222 |
| Average OMM | Residue | ASN12 | PLC | 0.038080633652616436 | 0.6590878901414395 | 0.057777778 |
| CS-OMM | Residue | ASN12 | PLM | 0.002021949 | 0.098365087 | 0.020555555555555525 |
| CS-OMM | Residue | ASN12 | PLC | 0.041989564567699145 | -0.429438729 | 0.097777778 |
| Average OMM | Residue | VAL13 | PLM | 0 |  | 0 |
| Average OMM | Residue | VAL13 | PLC | 0.00250548 | 0.45098636628430516 | 0.005555556 |
| CS-OMM | Residue | VAL13 | PLM | 0 |  | 0 |
| CS-OMM | Residue | VAL13 | PLC | 0.000123526 | 0.11117320932234838 | 0.001111111 |
| Average OMM | Residue | TRP14 | PLM | -0.000263186 | -0.157911564 | 0.001666667 |
| Average OMM | Residue | TRP14 | PLC | 0.000889507 | 0.5337039300692923 | 0.001666667 |
| CS-OMM | Residue | TRP14 | PLM | 0.000424508 | -0.084901539 | 0.005 |
| CS-OMM | Residue | TRP14 | PLC | 0.000223292 | -0.133975058 | 0.001666667 |
| Average OMM | Residue | GLY15 | PLM | -0.002858749 | -0.270828832 | 0.010555555555555549 |
| Average OMM | Residue | GLY15 | PLC | 0.027677626533944195 | 0.7016863064943609 | 0.039444444444444386 |
| CS-OMM | Residue | GLY15 | PLM | 0.005146537 | -0.140360113 | 0.036666667 |
| CS-OMM | Residue | GLY15 | PLC | 0.026542567012410267 | -0.482592127 | 0.055 |
| Average OMM | Residue | LYS16 | PLM | -0.005618409 | -0.259311204 | 0.021666666666666584 |
| Average OMM | Residue | LYS16 | PLC | 0.041265194474411605 | 0.7502762631711212 | 0.055 |
| CS-OMM | Residue | LYS16 | PLM | 0.013175155514680839 | 0.8469742830866287 | 0.015555555555555494 |
| CS-OMM | Residue | LYS16 | PLC | 0.011314301 | -0.185143106 | 0.061111111 |
| Average OMM | Residue | VAL17 | PLM | 0 |  | 0 |
| Average OMM | Residue | VAL17 | PLC | 0.000227219 | 0.40899439400687454 | 0.000555556 |
| CS-OMM | Residue | VAL17 | PLM | 0.00018867 | -0.084901539 | 0.002222222 |
| CS-OMM | Residue | VAL17 | PLC | 0 |  | 0 |
| Average OMM | Residue | GLU18 | PLM | -0.002456402 | -0.157911564 | 0.015555555555555501 |
| Average OMM | Residue | GLU18 | PLC | 0.030726986 | 0.6011801702971685 | 0.051111111 |
| CS-OMM | Residue | GLU18 | PLM | 0.015621684721084486 | -0.144200167 | 0.10833333333333324 |
| CS-OMM | Residue | GLU18 | PLC | 0.051879772 | -0.518797718 | 0.1 |
| Average OMM | Residue | ALA19 | PLM | -0.003888882 | -0.225806073 | 0.017222222 |
| Average OMM | Residue | ALA19 | PLC | 0.038914745736765385 | 0.6608164370394127 | 0.058888888888888845 |
| CS-OMM | Residue | ALA19 | PLM | 0.011894417673115039 | -0.135506024 | 0.087777778 |
| CS-OMM | Residue | ALA19 | PLC | 0.041862666 | -0.479954128 | 0.087222222 |
| Average OMM | Residue | ASP20 | PLM | -0.001554874 | -0.174923322 | 0.008888889 |
| Average OMM | Residue | ASP20 | PLC | 0.016909256139326177 | 0.6917422966087999 | 0.024444444444444383 |
| CS-OMM | Residue | ASP20 | PLM | 0.002012951 | -0.054898675 | 0.036666667 |
| CS-OMM | Residue | ASP20 | PLC | 0.035060908 | -0.460654266 | 0.076111111 |
| Average OMM | Residue | LEU21 | PLM | -0.001742665 | -0.165094613 | 0.010555556 |
| Average OMM | Residue | LEU21 | PLC | 0.005678468 | 0.5379601374320218 | 0.010555555555555549 |
| CS-OMM | Residue | LEU21 | PLM | 0.008366917 | -0.111558891 | 0.075 |
| CS-OMM | Residue | LEU21 | PLC | 0.019054348762989275 | -0.428722847 | 0.044444444 |
| Average OMM | Residue | ALA22 | PLM | -0.001099547 | -0.16493212 | 0.006666667 |
| Average OMM | Residue | ALA22 | PLC | 0.004227628 | 0.5853638379997514 | 0.007222222 |
| CS-OMM | Residue | ALA22 | PLM | 0.004400653 | -0.123768356 | 0.035555556 |
| CS-OMM | Residue | ALA22 | PLC | 0.007329265 | -0.299833564 | 0.024444444444444383 |
| Average OMM | Residue | GLY23 | PLM | 0 |  | 0 |
| Average OMM | Residue | GLY23 | PLC | 0 |  | 0 |
| CS-OMM | Residue | GLY23 | PLM | 9.43350435813023e-05 | -0.084901539 | 0.001111111 |
| CS-OMM | Residue | GLY23 | PLC | 0.002520899 | -0.216077067 | 0.011666666666666638 |
| Average OMM | Residue | HSD24 | PLM | 0 |  | 0 |
| Average OMM | Residue | HSD24 | PLC | 0.000908876 | 0.40899439400687454 | 0.002222222 |
| CS-OMM | Residue | HSD24 | PLM | 0 |  | 0 |
| CS-OMM | Residue | HSD24 | PLC | 0 |  | 0 |
| Average OMM | Residue | GLY25 | PLM | 0 |  | 0 |
| Average OMM | Residue | GLY25 | PLC | 0 |  | 0 |
| CS-OMM | Residue | GLY25 | PLM | 0 |  | 0 |
| CS-OMM | Residue | GLY25 | PLC | 0 |  | 0 |
| Average OMM | Residue | GLN26 | PLM | -8.77E-05 | -0.157911564 | 0.000555556 |
| Average OMM | Residue | GLN26 | PLC | 0 |  | 0 |
| CS-OMM | Residue | GLN26 | PLM | 0.000517681 | 0.1553041749766269 | 0.003333333 |
| CS-OMM | Residue | GLN26 | PLC | 0 |  | 0 |
| Average OMM | Residue | GLU27 | PLM | 0 |  | 0 |
| Average OMM | Residue | GLU27 | PLC | 0 |  | 0 |
| CS-OMM | Residue | GLU27 | PLM | 0.00914569 | 0.15530417497662694 | 0.058888889 |
| CS-OMM | Residue | GLU27 | PLC | 0 |  | 0 |
| Average OMM | Residue | VAL28 | PLM | 0 |  | 0 |
| Average OMM | Residue | VAL28 | PLC | 0 |  | 0 |
| CS-OMM | Residue | VAL28 | PLM | 0.00025884 | 0.1553041749766269 | 0.001666667 |
| CS-OMM | Residue | VAL28 | PLC | 0 |  | 0 |
| Average OMM | Residue | LEU29 | PLM | 0 |  | 0 |
| Average OMM | Residue | LEU29 | PLC | 0 |  | 0 |
| CS-OMM | Residue | LEU29 | PLM | 0 |  | 0 |
| CS-OMM | Residue | LEU29 | PLC | 0 |  | 0 |
| Average OMM | Residue | ILE30 | PLM | 0 |  | 0 |
| Average OMM | Residue | ILE30 | PLC | 0 |  | 0 |
| CS-OMM | Residue | ILE30 | PLM | 0.003019803 | 0.15530417497662685 | 0.019444444444444417 |
| CS-OMM | Residue | ILE30 | PLC | 0 |  | 0 |
| Average OMM | Residue | GLY31 | PLM | 0 |  | 0 |
| Average OMM | Residue | GLY31 | PLC | 0 |  | 0 |
| CS-OMM | Residue | GLY31 | PLM | 0.002847243 | 0.15530417497662682 | 0.018333333333333333 |
| CS-OMM | Residue | GLY31 | PLC | 0 |  | 0 |
| Average OMM | Residue | LEU32 | PLM | 0 |  | 0 |
| Average OMM | Residue | LEU32 | PLC | 0 |  | 0 |
| CS-OMM | Residue | LEU32 | PLM | 0.001121641 | 0.1553041749766269 | 0.007222222 |
| CS-OMM | Residue | LEU32 | PLC | 0 |  | 0 |
| Average OMM | Residue | PHE33 | PLM | 0 |  | 0 |
| Average OMM | Residue | PHE33 | PLC | 0 |  | 0 |
| CS-OMM | Residue | PHE33 | PLM | 0.000611347 | 0.10003853868199149 | 0.006111111 |
| CS-OMM | Residue | PHE33 | PLC | 0 |  | 0 |
| Average OMM | Residue | LYS34 | PLM | 0.003290784 | 0.32907841149500644 | 0.01 |
| Average OMM | Residue | LYS34 | PLC | -0.000534768 | -0.320860971 | 0.001666667 |
| CS-OMM | Residue | LYS34 | PLM | 0.012173174983351234 | 0.11010912045242333 | 0.11055555555555545 |
| CS-OMM | Residue | LYS34 | PLC | 0 |  | 0 |
| Average OMM | Residue | THR35 | PLM | 0.002451042 | 0.735312526 | 0.003333333 |
| Average OMM | Residue | THR35 | PLC | -6.19E-05 | -0.111362995 | 0.000555556 |
| CS-OMM | Residue | THR35 | PLM | 0.003310002 | 0.039720021386150155 | 0.083333333 |
| CS-OMM | Residue | THR35 | PLC | 0 |  | 0 |
| Average OMM | Residue | HSD36 | PLM | 0.000736019 | 0.6624167968706918 | 0.001111111 |
| Average OMM | Residue | HSD36 | PLC | -6.19E-05 | -0.111362995 | 0.000555556 |
| CS-OMM | Residue | HSD36 | PLM | 0.000217425 | 0.003403179 | 0.063888889 |
| CS-OMM | Residue | HSD36 | PLC | 0 |  | 0 |
| Average OMM | Residue | PRO37 | PLM | -0.000430726 | -0.077530691 | 0.005555556 |
| Average OMM | Residue | PRO37 | PLC | -6.19E-05 | -0.111362995 | 0.000555556 |
| CS-OMM | Residue | PRO37 | PLM | 0.000795637 | -0.010157062 | 0.078333333 |
| CS-OMM | Residue | PRO37 | PLC | 0 |  | 0 |
| Average OMM | Residue | GLU38 | PLM | -0.003214767 | -0.222560781 | 0.014444444444444418 |
| Average OMM | Residue | GLU38 | PLC | -0.000247473 | -0.111362995 | 0.002222222 |
| CS-OMM | Residue | GLU38 | PLM | 0.013715098164728348 | -0.096059053 | 0.1427777777777777 |
| CS-OMM | Residue | GLU38 | PLC | 0.001845487 | -0.17483561 | 0.010555555555555549 |
| Average OMM | Residue | THR39 | PLM | 0 |  | 0 |
| Average OMM | Residue | THR39 | PLC | 0 |  | 0 |
| CS-OMM | Residue | THR39 | PLM | 0.002883578 | -0.049432762 | 0.058333333 |
| CS-OMM | Residue | THR39 | PLC | 0 |  | 0 |
| Average OMM | Residue | LEU40 | PLM | 0 |  | 0 |
| Average OMM | Residue | LEU40 | PLC | 0 |  | 0 |
| CS-OMM | Residue | LEU40 | PLM | 0.000125124 | -0.075074459 | 0.001666667 |
| CS-OMM | Residue | LEU40 | PLC | 0 |  | 0 |
| Average OMM | Residue | ASP41 | PLM | -0.003131541 | -0.187892468 | 0.016666667 |
| Average OMM | Residue | ASP41 | PLC | 0 |  | 0 |
| CS-OMM | Residue | ASP41 | PLM | 0.013249108287914878 | -0.141114763 | 0.093888889 |
| CS-OMM | Residue | ASP41 | PLC | 0.003172472 | -0.184208041 | 0.017222222222222222 |
| Average OMM | Residue | LYS42 | PLM | 0.001800896 | 0.20260077023481127 | 0.008888889 |
| Average OMM | Residue | LYS42 | PLC | 0 |  | 0 |
| CS-OMM | Residue | LYS42 | PLM | 0.015788196928712854 | -0.113675018 | 0.1388888888888888 |
| CS-OMM | Residue | LYS42 | PLC | 0.008085026 | -0.177476179 | 0.045555556 |
| Average OMM | Residue | PHE43 | PLM | 0 |  | 0 |
| Average OMM | Residue | PHE43 | PLC | 0 |  | 0 |
| CS-OMM | Residue | PHE43 | PLM | 0.005137272 | -0.111410728 | 0.046111111 |
| CS-OMM | Residue | PHE43 | PLC | 0.000307893 | -0.184735582 | 0.001666667 |
| Average OMM | Residue | ASP44 | PLM | 0.008925835 | 0.4868637266390019 | 0.018333333 |
| Average OMM | Residue | ASP44 | PLC | 0 |  | 0 |
| CS-OMM | Residue | ASP44 | PLM | 0.007832822 | -0.05923983 | 0.1322222222222221 |
| CS-OMM | Residue | ASP44 | PLC | 0.006328038 | -0.189841144 | 0.033333333333333326 |
| Average OMM | Residue | LYS45 | PLM | 0.005232891 | 0.4957476041828488 | 0.010555556 |
| Average OMM | Residue | LYS45 | PLC | 0 |  | 0 |
| CS-OMM | Residue | LYS45 | PLM | 0.00113433 | -0.040035165 | 0.028333333333333318 |
| CS-OMM | Residue | LYS45 | PLC | 0.000260722 | 0.021331819 | 0.012222222222222223 |
| Average OMM | Residue | PHE46 | PLM | 0 |  | 0 |
| Average OMM | Residue | PHE46 | PLC | 0 |  | 0 |
| CS-OMM | Residue | PHE46 | PLM | 0.000680843 | -0.111410728 | 0.006111111 |
| CS-OMM | Residue | PHE46 | PLC | 0 |  | 0 |
| Average OMM | Residue | LYS47 | PLM | 0.00392518 | 0.19625899929318535 | 0.019999999999999917 |
| Average OMM | Residue | LYS47 | PLC | 0 |  | 0 |
| CS-OMM | Residue | LYS47 | PLM | 0.00912676 | -0.107373645 | 0.085 |
| CS-OMM | Residue | LYS47 | PLC | 0.007492054 | -0.184735582 | 0.040555555555555504 |
| Average OMM | Residue | ASN48 | PLM | 0.000380999 | 0.022859940072548574 | 0.016666666666666607 |
| Average OMM | Residue | ASN48 | PLC | -0.000321957 | -0.289760903 | 0.001111111 |
| CS-OMM | Residue | ASN48 | PLM | 0.001504811 | -0.031134029 | 0.048333333 |
| CS-OMM | Residue | ASN48 | PLC | 0.005131544 | -0.184735582 | 0.027777778 |
| Average OMM | Residue | LEU49 | PLM | 0 |  | 0 |
| Average OMM | Residue | LEU49 | PLC | 0 |  | 0 |
| CS-OMM | Residue | LEU49 | PLM | 0.000296878 | -0.066797499 | 0.004444444 |
| CS-OMM | Residue | LEU49 | PLC | 0 |  | 0 |
| Average OMM | Residue | LYS50 | PLM | -0.001188206 | -0.178230836 | 0.006666667 |
| Average OMM | Residue | LYS50 | PLC | -0.000482935 | -0.289760903 | 0.001666667 |
| CS-OMM | Residue | LYS50 | PLM | 0.003201229 | 0.045731845 | 0.07 |
| CS-OMM | Residue | LYS50 | PLC | 0.001436832 | -0.184735582 | 0.007777778 |
| Average OMM | Residue | SER51 | PLM | -0.000759427 | -0.12426989 | 0.006111111 |
| Average OMM | Residue | SER51 | PLC | -0.001931739 | -0.289760903 | 0.006666667 |
| CS-OMM | Residue | SER51 | PLM | 0.012437888613928414 | 0.14632810134033447 | 0.085 |
| CS-OMM | Residue | SER51 | PLC | 0 |  | 0 |
| Average OMM | Residue | GLU52 | PLM | 0.005118998 | 0.32907841149500655 | 0.015555555555555501 |
| Average OMM | Residue | GLU52 | PLC | -0.002212902 | -0.33193531 | 0.006666667 |
| CS-OMM | Residue | GLU52 | PLM | 0.013431089 | 0.1667307630315626 | 0.080555556 |
| CS-OMM | Residue | GLU52 | PLC | 0 |  | 0 |
| Average OMM | Residue | GLU53 | PLM | 0.002873853 | 0.10557011203718501 | 0.027222222222222165 |
| Average OMM | Residue | GLU53 | PLC | -0.014732568 | -0.509973508 | 0.028888889 |
| CS-OMM | Residue | GLU53 | PLM | 0.02720908 | 0.14490042584215687 | 0.18777777777777768 |
| CS-OMM | Residue | GLU53 | PLC | 0.000308814 | 0.11117320932234839 | 0.002777778 |
| Average OMM | Residue | ASP54 | PLM | -0.001740759 | -0.15666831 | 0.011111111111111084 |
| Average OMM | Residue | ASP54 | PLC | -0.000804891 | -0.289760903 | 0.002777778 |
| CS-OMM | Residue | ASP54 | PLM | 0.006693618 | 0.071292977 | 0.093888889 |
| CS-OMM | Residue | ASP54 | PLC | 0 |  | 0 |
| Average OMM | Residue | MET55 | PLM | 0 |  | 0 |
| Average OMM | Residue | MET55 | PLC | 0 |  | 0 |
| CS-OMM | Residue | MET55 | PLM | 8.628009720923702e-05 | 0.1553041749766268 | 0.000555556 |
| CS-OMM | Residue | MET55 | PLC | 0 |  | 0 |
| Average OMM | Residue | LYS56 | PLM | 0.001178889 | 0.30314281552300837 | 0.003888889 |
| Average OMM | Residue | LYS56 | PLC | -0.001495997 | -0.448799201 | 0.003333333 |
| CS-OMM | Residue | LYS56 | PLM | 0.017925674055035163 | 0.1689330539217976 | 0.10611111111111095 |
| CS-OMM | Residue | LYS56 | PLC | 0.000672975 | -0.403784881 | 0.001666667 |
| Average OMM | Residue | GLY57 | PLM | -0.001361414 | -0.204212143 | 0.006666667 |
| Average OMM | Residue | GLY57 | PLC | 0.000402169 | 0.3619523491310563 | 0.001111111 |
| CS-OMM | Residue | GLY57 | PLM | 0.002916847 | -0.070004326 | 0.041666666666666574 |
| CS-OMM | Residue | GLY57 | PLC | 0.002528323 | -0.350075461 | 0.007222222 |
| Average OMM | Residue | SER58 | PLM | -0.00071655 | -0.184255742 | 0.003888889 |
| Average OMM | Residue | SER58 | PLC | 0 |  | 0 |
| CS-OMM | Residue | SER58 | PLM | 0.0005316 | -0.079740068 | 0.006666667 |
| CS-OMM | Residue | SER58 | PLC | 0.000672975 | -0.403784881 | 0.001666667 |
| Average OMM | Residue | GLU59 | PLM | -0.008280867 | -0.252636624 | 0.032777777777777746 |
| Average OMM | Residue | GLU59 | PLC | 0.015266471814769008 | 0.5184839484261192 | 0.029444444444444332 |
| CS-OMM | Residue | GLU59 | PLM | 0.030861562927770952 | -0.172518054 | 0.17888888888888868 |
| CS-OMM | Residue | GLU59 | PLC | 0.026660566028658645 | -0.366328388 | 0.072777778 |
| Average OMM | Residue | ASP60 | PLM | -0.001469421 | -0.220413085 | 0.006666667 |
| Average OMM | Residue | ASP60 | PLC | 0.000236266 | 0.4252781787715809 | 0.000555556 |
| CS-OMM | Residue | ASP60 | PLM | 0.014725594816339798 | -0.185357138 | 0.079444444 |
| CS-OMM | Residue | ASP60 | PLC | 0.003132085 | -0.331632579 | 0.009444444 |
| Average OMM | Residue | LEU61 | PLM | 0 |  | 0 |
| Average OMM | Residue | LEU61 | PLC | 0 |  | 0 |
| CS-OMM | Residue | LEU61 | PLM | 0 |  | 0 |
| CS-OMM | Residue | LEU61 | PLC | 0 |  | 0 |
| Average OMM | Residue | LYS62 | PLM | -0.001473786 | -0.176854314 | 0.008333333 |
| Average OMM | Residue | LYS62 | PLC | 0.002871843 | 0.5743686564001388 | 0.005 |
| CS-OMM | Residue | LYS62 | PLM | 0.009172298 | -0.160292594 | 0.057222222222222084 |
| CS-OMM | Residue | LYS62 | PLC | 0.006784225 | -0.393922761 | 0.017222222222222215 |
| Average OMM | Residue | LYS63 | PLM | -0.003838458 | -0.177159596 | 0.021666666666666584 |
| Average OMM | Residue | LYS63 | PLC | 0.013688402157192293 | 0.5133150808947121 | 0.026666666666666606 |
| CS-OMM | Residue | LYS63 | PLM | 0.025560238305938933 | -0.174936992 | 0.146111111 |
| CS-OMM | Residue | LYS63 | PLC | 0.020629472 | -0.386802602 | 0.053333333 |
| Average OMM | Residue | HSE64 | PLM | -3.87E-05 | -0.06971365 | 0.000555556 |
| Average OMM | Residue | HSE64 | PLC | 0 |  | 0 |
| CS-OMM | Residue | HSE64 | PLM | 0.000330173 | -0.084901539 | 0.003888889 |
| CS-OMM | Residue | HSE64 | PLC | 0.00018424 | -0.331632579 | 0.000555556 |
| Average OMM | Residue | GLY65 | PLM | 0 |  | 0 |
| Average OMM | Residue | GLY65 | PLC | 0 |  | 0 |
| CS-OMM | Residue | GLY65 | PLM | 0 |  | 0 |
| CS-OMM | Residue | GLY65 | PLC | 0 |  | 0 |
| Average OMM | Residue | CYS66 | PLM | -0.001768003 | -0.144654764 | 0.012222222222222218 |
| Average OMM | Residue | CYS66 | PLC | 0.005918267 | 0.5326440280438489 | 0.011111111111111108 |
| CS-OMM | Residue | CYS66 | PLM | 0.00789672 | -0.111922018 | 0.070555556 |
| CS-OMM | Residue | CYS66 | PLC | 0.014591060817932688 | -0.410373586 | 0.035555555555555465 |
| Average OMM | Residue | THR67 | PLM | -0.000542217 | -0.06971365 | 0.007777778 |
| Average OMM | Residue | THR67 | PLC | 0.000603254 | 0.36195234913105623 | 0.001666667 |
| CS-OMM | Residue | THR67 | PLM | 0.009724177 | -0.13891681 | 0.07 |
| CS-OMM | Residue | THR67 | PLC | 0.004297274 | -0.407110174 | 0.010555555555555549 |
| Average OMM | Residue | VAL68 | PLM | 0 |  | 0 |
| Average OMM | Residue | VAL68 | PLC | 0 |  | 0 |
| CS-OMM | Residue | VAL68 | PLM | 0 |  | 0 |
| CS-OMM | Residue | VAL68 | PLC | 0 |  | 0 |
| Average OMM | Residue | LEU69 | PLM | 0 |  | 0 |
| Average OMM | Residue | LEU69 | PLC | 0 |  | 0 |
| CS-OMM | Residue | LEU69 | PLM | 0 |  | 0 |
| CS-OMM | Residue | LEU69 | PLC | 0 |  | 0 |
| Average OMM | Residue | THR70 | PLM | -0.000542217 | -0.06971365 | 0.007777778 |
| Average OMM | Residue | THR70 | PLC | 0.005663447 | 0.4633729235310989 | 0.012222222222222192 |
| CS-OMM | Residue | THR70 | PLM | 0.011511539338277083 | -0.140957625 | 0.081666667 |
| CS-OMM | Residue | THR70 | PLC | 0.019950364 | -0.52044429 | 0.038333333333333254 |
| Average OMM | Residue | ALA71 | PLM | 0 |  | 0 |
| Average OMM | Residue | ALA71 | PLC | 0 |  | 0 |
| CS-OMM | Residue | ALA71 | PLM | 0.00018867 | -0.084901539 | 0.002222222 |
| CS-OMM | Residue | ALA71 | PLC | 0.001415248 | -0.36392079 | 0.003888889 |
| Average OMM | Residue | LEU72 | PLM | 0 |  | 0 |
| Average OMM | Residue | LEU72 | PLC | 0 |  | 0 |
| CS-OMM | Residue | LEU72 | PLM | 0 |  | 0 |
| CS-OMM | Residue | LEU72 | PLC | 0 |  | 0 |
| Average OMM | Residue | GLY73 | PLM | 0 |  | 0 |
| Average OMM | Residue | GLY73 | PLC | 0 |  | 0 |
| CS-OMM | Residue | GLY73 | PLM | 0 |  | 0 |
| CS-OMM | Residue | GLY73 | PLC | 0.000736961 | -0.331632579 | 0.002222222 |
| Average OMM | Residue | THR74 | PLM | 0 |  | 0 |
| Average OMM | Residue | THR74 | PLC | 0.001877046 | 0.5631137349223587 | 0.003333333 |
| CS-OMM | Residue | THR74 | PLM | 0.008628002 | -0.127298388 | 0.067777778 |
| CS-OMM | Residue | THR74 | PLC | 0.015020879791462398 | -0.429167994 | 0.035 |
| Average OMM | Residue | ILE75 | PLM | 0 |  | 0 |
| Average OMM | Residue | ILE75 | PLC | 0 |  | 0 |
| CS-OMM | Residue | ILE75 | PLM | 0.000141503 | -0.084901539 | 0.001666667 |
| CS-OMM | Residue | ILE75 | PLC | 0 |  | 0 |
| Average OMM | Residue | LEU76 | PLM | 0 |  | 0 |
| Average OMM | Residue | LEU76 | PLC | 0 |  | 0 |
| CS-OMM | Residue | LEU76 | PLM | 0 |  | 0 |
| CS-OMM | Residue | LEU76 | PLC | 0 |  | 0 |
| Average OMM | Residue | LYS77 | PLM | -0.001648444 | -0.17454112 | 0.009444444 |
| Average OMM | Residue | LYS77 | PLC | 0.017819975417768315 | 0.5173541250319851 | 0.034444444444444326 |
| CS-OMM | Residue | LYS77 | PLM | 0.014907912511132661 | -0.162631773 | 0.091666667 |
| CS-OMM | Residue | LYS77 | PLC | 0.041925458 | -0.446543339 | 0.093888889 |
| Average OMM | Residue | LYS78 | PLM | -0.003235287 | -0.102166955 | 0.031666667 |
| Average OMM | Residue | LYS78 | PLC | 0.011416016 | 0.5268930449730358 | 0.021666666666666636 |
| CS-OMM | Residue | LYS78 | PLM | 0.028181200279262873 | -0.225449602 | 0.12499999999999993 |
| CS-OMM | Residue | LYS78 | PLC | 0.01686624 | -0.157301718 | 0.10722222222222218 |
| Average OMM | Residue | LYS79 | PLM | -0.018908493 | -0.25590441 | 0.073888889 |
| Average OMM | Residue | LYS79 | PLC | 0.034302770240021825 | 0.6639245852907473 | 0.051666667 |
| CS-OMM | Residue | LYS79 | PLM | 0.03972199 | -0.265797703 | 0.14944444444444438 |
| CS-OMM | Residue | LYS79 | PLC | 0.023899988 | 0.1265293497307644 | 0.1888888888888888 |
| Average OMM | Residue | GLY80 | PLM | -0.003643653 | -0.105783487 | 0.034444444 |
| Average OMM | Residue | GLY80 | PLC | 0.002330505 | 0.27966054000913776 | 0.008333333 |
| CS-OMM | Residue | GLY80 | PLM | 0.027024571401736204 | -0.233866483 | 0.11555555555555545 |
| CS-OMM | Residue | GLY80 | PLC | 0.05207765 | 0.55467319 | 0.093888889 |
| Average OMM | Residue | GLN81 | PLM | 0.007241497 | 0.102635393 | 0.070555556 |
| Average OMM | Residue | GLN81 | PLC | 0.003742908 | 0.2929232013844207 | 0.012777777777777758 |
| CS-OMM | Residue | GLN81 | PLM | 0.035019562 | -0.290484848 | 0.12055555555555551 |
| CS-OMM | Residue | GLN81 | PLC | 0.044080475 | 0.33620701457506996 | 0.13111111111111107 |
| Average OMM | Residue | HSD82 | PLM | -0.000911943 | -0.074613534 | 0.012222222222222167 |
| Average OMM | Residue | HSD82 | PLC | 0 |  | 0 |
| CS-OMM | Residue | HSD82 | PLM | 0.000888485 | -0.123020999 | 0.007222222 |
| CS-OMM | Residue | HSD82 | PLC | 0.009935815 | 0.6387309895173953 | 0.015555556 |
| Average OMM | Residue | ALA83 | PLM | 0.001376253 | 0.41287601751104314 | 0.003333333 |
| Average OMM | Residue | ALA83 | PLC | 0 |  | 0 |
| CS-OMM | Residue | ALA83 | PLM | 0.004193141 | -0.20965703 | 0.019999999999999917 |
| CS-OMM | Residue | ALA83 | PLC | 0.020185494725254622 | 0.6488194733117563 | 0.031111111111111086 |
| Average OMM | Residue | ALA84 | PLM | -0.001144947 | -0.103045205 | 0.011111111111111108 |
| Average OMM | Residue | ALA84 | PLC | -0.000559127 | -0.201285673 | 0.002777778 |
| CS-OMM | Residue | ALA84 | PLM | 0.012897983363277257 | -0.21105791 | 0.061111111 |
| CS-OMM | Residue | ALA84 | PLC | 0.030038547 | 0.6361104143191492 | 0.047222222 |
| Average OMM | Residue | GLU85 | PLM | -0.002038485 | -0.099169553 | 0.020555555555555525 |
| Average OMM | Residue | GLU85 | PLC | 0.000945063 | 0.4252781787715809 | 0.002222222 |
| CS-OMM | Residue | GLU85 | PLM | 0.016680575199559015 | -0.184202671 | 0.090555556 |
| CS-OMM | Residue | GLU85 | PLC | 0.009789289 | 0.24473222778647866 | 0.04 |
| Average OMM | Residue | ILE86 | PLM | 0 |  | 0 |
| Average OMM | Residue | ILE86 | PLC | 0 |  | 0 |
| CS-OMM | Residue | ILE86 | PLM | 0 |  | 0 |
| CS-OMM | Residue | ILE86 | PLC | 0 |  | 0 |
| Average OMM | Residue | GLN87 | PLM | 0.002827406 | 0.26785952758646553 | 0.010555555555555539 |
| Average OMM | Residue | GLN87 | PLC | -0.001341904 | -0.201285673 | 0.006666667 |
| CS-OMM | Residue | GLN87 | PLM | 0.013305926449775438 | -0.184235905 | 0.072222222 |
| CS-OMM | Residue | GLN87 | PLC | 0.018289139500976374 | 0.5675939845130619 | 0.032222222 |
| Average OMM | Residue | PRO88 | PLM | 0.011373349830588105 | 0.4094405939011726 | 0.027777778 |
| Average OMM | Residue | PRO88 | PLC | -0.000111825 | -0.201285673 | 0.000555556 |
| CS-OMM | Residue | PRO88 | PLM | 0.002261916 | -0.056547904 | 0.039999999999999876 |
| CS-OMM | Residue | PRO88 | PLC | 0.005348482 | 0.2831549431467362 | 0.018888888888888834 |
| Average OMM | Residue | LEU89 | PLM | 0.001377077 | 0.49574760418284886 | 0.002777778 |
| Average OMM | Residue | LEU89 | PLC | 0 |  | 0 |
| CS-OMM | Residue | LEU89 | PLM | 0.000206 | -0.074159901 | 0.002777778 |
| CS-OMM | Residue | LEU89 | PLC | 0.000203996 | -0.183596845 | 0.001111111 |
| Average OMM | Residue | ALA90 | PLM | 0.009364121 | 0.4957476041828489 | 0.018888888888888834 |
| Average OMM | Residue | ALA90 | PLC | 0 |  | 0 |
| CS-OMM | Residue | ALA90 | PLM | 0 |  | 0 |
| CS-OMM | Residue | ALA90 | PLC | 0 |  | 0 |
| Average OMM | Residue | GLN91 | PLM | 0.019424363163319715 | 0.4924486435771196 | 0.039444444 |
| Average OMM | Residue | GLN91 | PLC | -0.002236507 | -0.201285673 | 0.011111111111111084 |
| CS-OMM | Residue | GLN91 | PLM | 0.009934982 | -0.126829555 | 0.078333333 |
| CS-OMM | Residue | GLN91 | PLC | 0.005053652 | -0.055466915 | 0.091111111 |
| Average OMM | Residue | SER92 | PLM | 0.012455251016304555 | 0.4670719131114219 | 0.026666666666666606 |
| Average OMM | Residue | SER92 | PLC | 0 |  | 0 |
| CS-OMM | Residue | SER92 | PLM | 0.000238878 | 0.017915814569715614 | 0.013333333333333275 |
| CS-OMM | Residue | SER92 | PLC | 0.009540707 | 0.4641425083656179 | 0.020555555555555525 |
| Average OMM | Residue | HSD93 | PLM | 0.000550831 | 0.4957476041828491 | 0.001111111 |
| Average OMM | Residue | HSD93 | PLC | 0 |  | 0 |
| CS-OMM | Residue | HSD93 | PLM | 0 |  | 0 |
| CS-OMM | Residue | HSD93 | PLC | 0 |  | 0 |
| Average OMM | Residue | ALA94 | PLM | 0.014872428 | 0.495747604 | 0.029999999999999916 |
| Average OMM | Residue | ALA94 | PLC | 0 |  | 0 |
| CS-OMM | Residue | ALA94 | PLM | 0.000848503 | -0.017355747 | 0.048888889 |
| CS-OMM | Residue | ALA94 | PLC | 0.003810179 | -0.190508968 | 0.019999999999999973 |
| Average OMM | Residue | THR95 | PLM | 0.023210960851919083 | 0.49152622980534527 | 0.047222222 |
| Average OMM | Residue | THR95 | PLC | -0.000335476 | -0.201285673 | 0.001666667 |
| CS-OMM | Residue | THR95 | PLM | 0.006045417 | -0.047518564 | 0.12722222222222215 |
| CS-OMM | Residue | THR95 | PLC | 0.004137654 | -0.052449133 | 0.078888889 |
| Average OMM | Residue | LYS96 | PLM | 0.02422166 | 0.37912163709156477 | 0.063888889 |
| Average OMM | Residue | LYS96 | PLC | -0.001565555 | -0.201285673 | 0.007777778 |
| CS-OMM | Residue | LYS96 | PLM | 0.009434156 | -0.055314267 | 0.1705555555555555 |
| CS-OMM | Residue | LYS96 | PLC | 0.013340681821540357 | -0.108657137 | 0.12277777777777767 |
| Average OMM | Residue | HSD97 | PLM | 0.012870365677769089 | 0.49290762170179536 | 0.026111111111111085 |
| Average OMM | Residue | HSD97 | PLC | 0 |  | 0 |
| CS-OMM | Residue | HSD97 | PLM | 0.007076623 | -0.060369299 | 0.11722222222222207 |
| CS-OMM | Residue | HSD97 | PLC | 0.004938026 | -0.060057068 | 0.082222222 |
| Average OMM | Residue | LYS98 | PLM | 0.025939148644668966 | 0.44047610906041695 | 0.058888889 |
| Average OMM | Residue | LYS98 | PLC | -0.000111825 | -0.201285673 | 0.000555556 |
| CS-OMM | Residue | LYS98 | PLM | 0.013304071577516825 | -0.069211933 | 0.19222222222222207 |
| CS-OMM | Residue | LYS98 | PLC | 0.004123173 | -0.083390011 | 0.049444444 |
| Average OMM | Residue | ILE99 | PLM | 0 |  | 0 |
| Average OMM | Residue | ILE99 | PLC | 0 |  | 0 |
| CS-OMM | Residue | ILE99 | PLM | 0.000243552 | -0.00515757 | 0.047222222222222165 |
| CS-OMM | Residue | ILE99 | PLC | 0 |  | 0 |
| Average OMM | Residue | PRO100 | PLM | 0.004669373 | 0.6465285838952249 | 0.007222222 |
| Average OMM | Residue | PRO100 | PLC | 0 |  | 0 |
| CS-OMM | Residue | PRO100 | PLM | 0.001568176 | -0.022948911 | 0.068333333 |
| CS-OMM | Residue | PRO100 | PLC | 0 |  | 0 |
| Average OMM | Residue | VAL101 | PLM | 0.023920606553663855 | 0.6624167968706918 | 0.036111111 |
| Average OMM | Residue | VAL101 | PLC | 0 |  | 0 |
| CS-OMM | Residue | VAL101 | PLM | 2.292253395433525e-05 | -0.00515757 | 0.004444444 |
| CS-OMM | Residue | VAL101 | PLC | 0 |  | 0 |
| Average OMM | Residue | LYS102 | PLM | 0.025024634548448332 | 0.6624167968706917 | 0.037777778 |
| Average OMM | Residue | LYS102 | PLC | -0.000494947 | -0.111362995 | 0.004444444 |
| CS-OMM | Residue | LYS102 | PLM | 0.003276819 | -0.036409104 | 0.09 |
| CS-OMM | Residue | LYS102 | PLC | 1.847283515875525e-05 | 0.033251103 | 0.000555556 |
| Average OMM | Residue | TYR103 | PLM | 0.003184312 | 0.6368623140484769 | 0.005 |
| Average OMM | Residue | TYR103 | PLC | -0.000185605 | -0.111362995 | 0.001666667 |
| CS-OMM | Residue | TYR103 | PLM | 0.004554388 | -0.051236864 | 0.088888889 |
| CS-OMM | Residue | TYR103 | PLC | 0 |  | 0 |
| Average OMM | Residue | LEU104 | PLM | 0 |  | 0 |
| Average OMM | Residue | LEU104 | PLC | 0 |  | 0 |
| CS-OMM | Residue | LEU104 | PLM | 0 |  | 0 |
| CS-OMM | Residue | LEU104 | PLC | 0 |  | 0 |
| Average OMM | Residue | GLU105 | PLM | 0.024656625 | 0.6624167968706919 | 0.037222222222222164 |
| Average OMM | Residue | GLU105 | PLC | 0 |  | 0 |
| CS-OMM | Residue | GLU105 | PLM | 9.169013581734062e-05 | -0.00515757 | 0.017777778 |
| CS-OMM | Residue | GLU105 | PLC | 0 |  | 0 |
| Average OMM | Residue | PHE106 | PLM | 0.009936252 | 0.6624167968706919 | 0.014999999999999916 |
| Average OMM | Residue | PHE106 | PLC | -6.19E-05 | -0.111362995 | 0.000555556 |
| CS-OMM | Residue | PHE106 | PLM | 0.000247328 | 0.022259554545630056 | 0.011111111111111084 |
| CS-OMM | Residue | PHE106 | PLC | 0 |  | 0 |
| Average OMM | Residue | ILE107 | PLM | 0 |  | 0 |
| Average OMM | Residue | ILE107 | PLC | 0 |  | 0 |
| CS-OMM | Residue | ILE107 | PLM | 0 |  | 0 |
| CS-OMM | Residue | ILE107 | PLC | 0 |  | 0 |
| Average OMM | Residue | SER108 | PLM | 0 |  | 0 |
| Average OMM | Residue | SER108 | PLC | 0 |  | 0 |
| CS-OMM | Residue | SER108 | PLM | 0 |  | 0 |
| CS-OMM | Residue | SER108 | PLC | 0 |  | 0 |
| Average OMM | Residue | GLU109 | PLM | 0.022448569 | 0.6624167968706918 | 0.033888888888888836 |
| Average OMM | Residue | GLU109 | PLC | -0.00037121 | -0.111362995 | 0.003333333 |
| CS-OMM | Residue | GLU109 | PLM | 9.27167953879425e-06 | 0.000596037 | 0.015555556 |
| CS-OMM | Residue | GLU109 | PLC | 0 |  | 0 |
| Average OMM | Residue | ILE110 | PLM | 0 |  | 0 |
| Average OMM | Residue | ILE110 | PLC | 0 |  | 0 |
| CS-OMM | Residue | ILE110 | PLM | 0.002070722 | 0.1553041749766269 | 0.013333333333333334 |
| CS-OMM | Residue | ILE110 | PLC | 0 |  | 0 |
| Average OMM | Residue | ILE111 | PLM | 0 |  | 0 |
| Average OMM | Residue | ILE111 | PLC | 0 |  | 0 |
| CS-OMM | Residue | ILE111 | PLM | 0 |  | 0 |
| CS-OMM | Residue | ILE111 | PLC | 0 |  | 0 |
| Average OMM | Residue | ILE112 | PLM | 0 |  | 0 |
| Average OMM | Residue | ILE112 | PLC | 0 |  | 0 |
| CS-OMM | Residue | ILE112 | PLM | 0 |  | 0 |
| CS-OMM | Residue | ILE112 | PLC | 0 |  | 0 |
| Average OMM | Residue | GLU113 | PLM | 0.011438194855491112 | 0.7353125264244289 | 0.015555556 |
| Average OMM | Residue | GLU113 | PLC | -0.00037121 | -0.111362995 | 0.003333333 |
| CS-OMM | Residue | GLU113 | PLM | 0.003109952 | 0.15549761076538507 | 0.019999999999999973 |
| CS-OMM | Residue | GLU113 | PLC | 0 |  | 0 |
| Average OMM | Residue | VAL114 | PLM | 0 |  | 0 |
| Average OMM | Residue | VAL114 | PLC | 0 |  | 0 |
| CS-OMM | Residue | VAL114 | PLM | 0.001898162 | 0.15530417497662682 | 0.012222222222222167 |
| CS-OMM | Residue | VAL114 | PLC | 0 |  | 0 |
| Average OMM | Residue | LEU115 | PLM | 0.000182821 | 0.3290784114950064 | 0.000555556 |
| Average OMM | Residue | LEU115 | PLC | 0.000227219 | 0.40899439400687454 | 0.000555556 |
| CS-OMM | Residue | LEU115 | PLM | 0 |  | 0 |
| CS-OMM | Residue | LEU115 | PLC | 0 |  | 0 |
| Average OMM | Residue | LYS116 | PLM | 0.024487374642769313 | 0.4364086569998498 | 0.056111111 |
| Average OMM | Residue | LYS116 | PLC | -6.19E-05 | -0.111362995 | 0.000555556 |
| CS-OMM | Residue | LYS116 | PLM | 0.001898162 | 0.15530417497662682 | 0.012222222222222167 |
| CS-OMM | Residue | LYS116 | PLC | 0 |  | 0 |
| Average OMM | Residue | LYS117 | PLM | 0.016842213 | 0.3368442586660223 | 0.05 |
| Average OMM | Residue | LYS117 | PLC | -0.000662669 | -0.04417791 | 0.014999999999999966 |
| CS-OMM | Residue | LYS117 | PLM | 0.013362500215114592 | 0.15928808203447864 | 0.083888889 |
| CS-OMM | Residue | LYS117 | PLC | 0 |  | 0 |
| Average OMM | Residue | ARG118 | PLM | 0.01578334 | 0.32655185468811776 | 0.048333333 |
| Average OMM | Residue | ARG118 | PLC | 0.001048494 | 0.048392052040637935 | 0.021666666666666667 |
| CS-OMM | Residue | ARG118 | PLM | 0.007977026 | 0.15778732131873957 | 0.050555556 |
| CS-OMM | Residue | ARG118 | PLC | 0.000177771 | -0.063997537 | 0.002777778 |
| Average OMM | Residue | HSD119 | PLM | 0.01686184 | 0.27099386250614976 | 0.062222222 |
| Average OMM | Residue | HSD119 | PLC | 0.006869277 | 0.19943063408734713 | 0.034444444444444416 |
| CS-OMM | Residue | HSD119 | PLM | 0.00651286 | 0.15630864427595959 | 0.041666667 |
| CS-OMM | Residue | HSD119 | PLC | 0.000453285 | -0.090656938 | 0.005 |
| Average OMM | Residue | SER120 | PLM | 0.017956015017911716 | 0.20987550020935786 | 0.085555556 |
| Average OMM | Residue | SER120 | PLC | 0.000891445 | 0.020311413800854546 | 0.043888889 |
| CS-OMM | Residue | SER120 | PLM | 0.040153978475806346 | 0.39495716533580044 | 0.10166666666666659 |
| CS-OMM | Residue | SER120 | PLC | 0.002745088 | 0.1029408125087388 | 0.026666666666666606 |
| Average OMM | Residue | GLY121 | PLM | 0.017578364 | 0.28764595682634125 | 0.061111111111111005 |
| Average OMM | Residue | GLY121 | PLC | 0.003120884 | 0.1605025870531759 | 0.019444444444444417 |
| CS-OMM | Residue | GLY121 | PLM | 0.019675395432397026 | 0.4118106020734266 | 0.047777777777777725 |
| CS-OMM | Residue | GLY121 | PLC | 0.001729361 | 0.11117320932234838 | 0.015555555555555501 |
| Average OMM | Residue | ASP122 | PLM | 0.015462742973027532 | 0.215759204 | 0.071666667 |
| Average OMM | Residue | ASP122 | PLC | 0.002142138 | 0.080330167 | 0.026666666666666606 |
| CS-OMM | Residue | ASP122 | PLM | 0.034986915 | 0.7587523747681438 | 0.046111111111111054 |
| CS-OMM | Residue | ASP122 | PLC | 0.001173495 | 0.1111732093223484 | 0.010555556 |
| Average OMM | Residue | PHE123 | PLM | 0.010480203175336428 | 0.3252476847518202 | 0.032222222 |
| Average OMM | Residue | PHE123 | PLC | 0.00467541 | 0.38253357700765833 | 0.012222222222222216 |
| CS-OMM | Residue | PHE123 | PLM | 0.0004314 | 0.1553041749766269 | 0.002777778 |
| CS-OMM | Residue | PHE123 | PLC | 0.000220626 | -0.198563095 | 0.001111111 |
| Average OMM | Residue | GLY124 | PLM | 0.000817014 | 0.735312526 | 0.001111111 |
| Average OMM | Residue | GLY124 | PLC | 0.005222076 | 0.19582786169658176 | 0.026666666666666523 |
| CS-OMM | Residue | GLY124 | PLM | 0.000776521 | 0.1553041749766269 | 0.005 |
| CS-OMM | Residue | GLY124 | PLC | 0.00476453 | 0.6597042058674425 | 0.007222222 |
| Average OMM | Residue | ALA125 | PLM | 0.004266159 | 0.3490493676437796 | 0.012222222222222192 |
| Average OMM | Residue | ALA125 | PLC | -0.003177977 | -0.065004081 | 0.048888888888888836 |
| CS-OMM | Residue | ALA125 | PLM | 0.001082399 | -0.216479766 | 0.005 |
| CS-OMM | Residue | ALA125 | PLC | 0.034201434 | 0.6549210753104756 | 0.052222222 |
| Average OMM | Residue | ASP126 | PLM | 0.005668585 | 0.1619595714149995 | 0.034999999999999906 |
| Average OMM | Residue | ASP126 | PLC | -0.003276871 | -0.143862631 | 0.022777777777777713 |
| CS-OMM | Residue | ASP126 | PLM | 0.006091601 | -0.23836699 | 0.025555555555555554 |
| CS-OMM | Residue | ASP126 | PLC | 0.067526921 | 0.6363793630808626 | 0.10611111111111099 |
| Average OMM | Residue | ALA127 | PLM | 0.005971611 | 0.7165933783137548 | 0.008333333 |
| Average OMM | Residue | ALA127 | PLC | -0.000157887 | -0.142098714 | 0.001111111 |
| CS-OMM | Residue | ALA127 | PLM | 0.002071444 | -0.310716551 | 0.006666667 |
| CS-OMM | Residue | ALA127 | PLC | 0.033873407 | 0.6486397167623913 | 0.052222222222222225 |
| Average OMM | Residue | GLN128 | PLM | 0.018473862 | 0.6650590498412923 | 0.027777777777777776 |
| Average OMM | Residue | GLN128 | PLC | -0.00037121 | -0.111362995 | 0.003333333 |
| CS-OMM | Residue | GLN128 | PLM | 0.003681396 | -0.28810928 | 0.012777777777777749 |
| CS-OMM | Residue | GLN128 | PLC | 0.045362593 | 0.6480370471979473 | 0.07 |
| Average OMM | Residue | GLY129 | PLM | 0.007578438 | 0.6820594298051842 | 0.011111111111111056 |
| Average OMM | Residue | GLY129 | PLC | 0 |  | 0 |
| CS-OMM | Residue | GLY129 | PLM | 8.628009720923702e-05 | 0.1553041749766268 | 0.000555556 |
| CS-OMM | Residue | GLY129 | PLC | 0.001802276 | 0.6488194733117563 | 0.002777778 |
| Average OMM | Residue | ALA130 | PLM | 0 |  | 0 |
| Average OMM | Residue | ALA130 | PLC | 0 |  | 0 |
| CS-OMM | Residue | ALA130 | PLM | 0 |  | 0 |
| CS-OMM | Residue | ALA130 | PLC | 0.000360455 | 0.6488194733117564 | 0.000555556 |
| Average OMM | Residue | MET131 | PLM | 0 |  | 0 |
| Average OMM | Residue | MET131 | PLC | 0 |  | 0 |
| CS-OMM | Residue | MET131 | PLM | 0 |  | 0 |
| CS-OMM | Residue | MET131 | PLC | 0 |  | 0 |
| Average OMM | Residue | SER132 | PLM | 0.004784121 | 0.6624167968706918 | 0.007222222 |
| Average OMM | Residue | SER132 | PLC | 0 |  | 0 |
| CS-OMM | Residue | SER132 | PLM | 0 |  | 0 |
| CS-OMM | Residue | SER132 | PLC | 0 |  | 0 |
| Average OMM | Residue | LYS133 | PLM | 0.026864681 | 0.6624167968706917 | 0.040555555555555504 |
| Average OMM | Residue | LYS133 | PLC | -6.19E-05 | -0.111362995 | 0.000555556 |
| CS-OMM | Residue | LYS133 | PLM | 0.006386951 | -0.310716551 | 0.020555556 |
| CS-OMM | Residue | LYS133 | PLC | 0.033161884191489736 | 0.6488194733117562 | 0.051111111 |
| Average OMM | Residue | ALA134 | PLM | 0.005152131 | 0.6624167968706915 | 0.007777778 |
| Average OMM | Residue | ALA134 | PLC | 0 |  | 0 |
| CS-OMM | Residue | ALA134 | PLM | 0 |  | 0 |
| CS-OMM | Residue | ALA134 | PLC | 0 |  | 0 |
| Average OMM | Residue | LEU135 | PLM | 0 |  | 0 |
| Average OMM | Residue | LEU135 | PLC | 0 |  | 0 |
| CS-OMM | Residue | LEU135 | PLM | 0 |  | 0 |
| CS-OMM | Residue | LEU135 | PLC | 0 |  | 0 |
| Average OMM | Residue | GLU136 | PLM | 0.032384821 | 0.6624167968706917 | 0.048888888888888836 |
| Average OMM | Residue | GLU136 | PLC | 0 |  | 0 |
| CS-OMM | Residue | GLU136 | PLM | 0.000345241 | -0.310716551 | 0.001111111 |
| CS-OMM | Residue | GLU136 | PLC | 0.004685918 | 0.6488194733117563 | 0.007222222 |
| Average OMM | Residue | LEU137 | PLM | 0.026128662543232817 | 0.6624167968706917 | 0.039444444444444414 |
| Average OMM | Residue | LEU137 | PLC | 0 |  | 0 |
| CS-OMM | Residue | LEU137 | PLM | 0.000517861 | -0.310716551 | 0.001666667 |
| CS-OMM | Residue | LEU137 | PLC | 0.012615934203284131 | 0.6488194733117563 | 0.019444444444444417 |
| Average OMM | Residue | PHE138 | PLM | 0 |  | 0 |
| Average OMM | Residue | PHE138 | PLC | 0 |  | 0 |
| CS-OMM | Residue | PHE138 | PLM | 0 |  | 0 |
| CS-OMM | Residue | PHE138 | PLC | 0 |  | 0 |
| Average OMM | Residue | ARG139 | PLM | 0.030544775 | 0.6624167968706917 | 0.046111111 |
| Average OMM | Residue | ARG139 | PLC | -0.000185605 | -0.111362995 | 0.001666667 |
| CS-OMM | Residue | ARG139 | PLM | 7.163291860729725e-05 | -0.00515757 | 0.013888888888888833 |
| CS-OMM | Residue | ARG139 | PLC | 0 |  | 0 |
| Average OMM | Residue | ASN140 | PLM | 0.032016812 | 0.6624167968706917 | 0.048333333 |
| Average OMM | Residue | ASN140 | PLC | -0.000123737 | -0.111362995 | 0.001111111 |
| CS-OMM | Residue | ASN140 | PLM | 5.730633488583788e-06 | -0.00515757 | 0.001111111 |
| CS-OMM | Residue | ASN140 | PLC | 0.028836421036078044 | 0.6488194733117564 | 0.044444444 |
| Average OMM | Residue | ASP141 | PLM | 0.013171724084372827 | 0.6585862042186433 | 0.02 |
| Average OMM | Residue | ASP141 | PLC | 0 |  | 0 |
| CS-OMM | Residue | ASP141 | PLM | 0 |  | 0 |
| CS-OMM | Residue | ASP141 | PLC | 0.015139121043940978 | 0.6488194733117562 | 0.023333333333333334 |
| Average OMM | Residue | ILE142 | PLM | 0 |  | 0 |
| Average OMM | Residue | ILE142 | PLC | 0 |  | 0 |
| CS-OMM | Residue | ILE142 | PLM | 0 |  | 0 |
| CS-OMM | Residue | ILE142 | PLC | 0 |  | 0 |
| Average OMM | Residue | ALA143 | PLM | 0.024656625 | 0.6624167968706919 | 0.037222222222222164 |
| Average OMM | Residue | ALA143 | PLC | 0 |  | 0 |
| CS-OMM | Residue | ALA143 | PLM | 1.1461266977167576e-05 | -0.00515757 | 0.002222222 |
| CS-OMM | Residue | ALA143 | PLC | 0 |  | 0 |
| Average OMM | Residue | ALA144 | PLM | 0.017296438584956934 | 0.6624167968706918 | 0.026111111111111085 |
| Average OMM | Residue | ALA144 | PLC | 0 |  | 0 |
| CS-OMM | Residue | ALA144 | PLM | 0.000815333 | -0.20965703 | 0.003888889 |
| CS-OMM | Residue | ALA144 | PLC | 0.018743674 | 0.6488194733117563 | 0.028888888888888836 |
| Average OMM | Residue | LYS145 | PLM | 0.002380298 | 0.5355670493415016 | 0.004444444 |
| Average OMM | Residue | LYS145 | PLC | -0.000335476 | -0.201285673 | 0.001666667 |
| CS-OMM | Residue | LYS145 | PLM | 0.007337996 | -0.20965703 | 0.035 |
| CS-OMM | Residue | LYS145 | PLC | 0.027975129481802124 | 0.6374080135094162 | 0.043888889 |
| Average OMM | Residue | TYR146 | PLM | 0 |  | 0 |
| Average OMM | Residue | TYR146 | PLC | 0 |  | 0 |
| CS-OMM | Residue | TYR146 | PLM | 9.129987027199235e-05 | -0.027389961 | 0.003333333 |
| CS-OMM | Residue | TYR146 | PLC | 0 |  | 0 |
| Average OMM | Residue | LYS147 | PLM | 0.048497214067416035 | 0.7335713052214197 | 0.066111111 |
| Average OMM | Residue | LYS147 | PLC | -0.000968007 | -0.217801645 | 0.004444444 |
| CS-OMM | Residue | LYS147 | PLM | 0.009225926 | -0.098264296 | 0.093888889 |
| CS-OMM | Residue | LYS147 | PLC | 0.020218541 | 0.6498816702372247 | 0.031111111 |
| Average OMM | Residue | GLU148 | PLM | 0.019985529137178437 | 0.6661843045726167 | 0.029999999999999905 |
| Average OMM | Residue | GLU148 | PLC | -0.00290746 | -0.201285673 | 0.014444444444444418 |
| CS-OMM | Residue | GLU148 | PLM | 0.017838487686716156 | -0.229351985 | 0.077777778 |
| CS-OMM | Residue | GLU148 | PLC | 0.065549267 | 0.6447468849604419 | 0.10166666666666653 |
| Average OMM | Residue | LEU149 | PLM | 0.022667823180807144 | 0.4800244908876813 | 0.047222222222222165 |
| Average OMM | Residue | LEU149 | PLC | -0.002683809 | -0.201285673 | 0.013333333333333334 |
| CS-OMM | Residue | LEU149 | PLM | 0.023547307 | -0.195323287 | 0.12055555555555549 |
| CS-OMM | Residue | LEU149 | PLC | 0.009719711 | -0.114349547 | 0.085 |
| Average OMM | Residue | GLY150 | PLM | 0.027421122 | 0.5250853166730886 | 0.052222222222222135 |
| Average OMM | Residue | GLY150 | PLC | -0.002012857 | -0.201285673 | 0.01 |
| CS-OMM | Residue | GLY150 | PLM | 0.020839502 | -0.128024245 | 0.1627777777777776 |
| CS-OMM | Residue | GLY150 | PLC | 0.010384739993341663 | -0.139496507 | 0.074444444 |
| Average OMM | Residue | PHE151 | PLM | 0.012353188529606615 | 0.517110218 | 0.023888888888888862 |
| Average OMM | Residue | PHE151 | PLC | 0 |  | 0 |
| CS-OMM | Residue | PHE151 | PLM | 0.008806636 | -0.061680716 | 0.1427777777777777 |
| CS-OMM | Residue | PHE151 | PLC | 0.009101353 | -0.162202337 | 0.056111111 |
| Average OMM | Residue | GLN152 | PLM | 0.040772209 | 0.7125240404949278 | 0.057222222 |
| Average OMM | Residue | GLN152 | PLC | -0.001044114 | -0.187940488 | 0.005555556 |
| CS-OMM | Residue | GLN152 | PLM | 0.016266713588609302 | -0.098586143 | 0.16499999999999995 |
| CS-OMM | Residue | GLN152 | PLC | 0.002727478 | -0.059871476 | 0.045555556 |
| Average OMM | Residue | GLY153 | PLM | 0.032946041671994934 | 0.6816422414895505 | 0.048333333 |
| Average OMM | Residue | GLY153 | PLC | -0.000989893 | -0.111362995 | 0.008888889 |
| CS-OMM | Residue | GLY153 | PLM | 0.009677435 | -0.074761297 | 0.12944444444444442 |
| CS-OMM | Residue | GLY153 | PLC | 0.006709956 | 0.12713600194973654 | 0.052777778 |
| Average OMM | AA type | ILE | PLM | 0 |  | 0 |
| Average OMM | AA type | ILE | PLC | 0 |  | 0 |
| CS-OMM | AA type | ILE | PLM | 0.007014291 | 0.085889276 | 0.081666667 |
| CS-OMM | AA type | ILE | PLC | 0 |  | 0 |
| Average OMM | AA type | LEU | PLM | 0.15630722381788784 | 0.752280756 | 0.20777777777777742 |
| Average OMM | AA type | LEU | PLC | 0.053432137 | 0.6542710685646432 | 0.081666667 |
| CS-OMM | AA type | LEU | PLM | 0.24821882893930863 | 0.5735480001165048 | 0.43277777777777615 |
| CS-OMM | AA type | LEU | PLC | 0.069468129 | 0.26718511324548516 | 0.25999999999999973 |
| Average OMM | AA type | VAL | PLM | 0.023920606553663855 | 0.6624167968706918 | 0.036111111 |
| Average OMM | AA type | VAL | PLC | 0.002731802 | 0.44702211169922007 | 0.006111111 |
| CS-OMM | AA type | VAL | PLM | 0.002835015 | 0.13791966904285635 | 0.020555556 |
| CS-OMM | AA type | VAL | PLC | 0.000123526 | 0.11117320932234838 | 0.001111111 |
| Average OMM | AA type | ALA | PLM | 0.15899700519688864 | 0.8888031346409934 | 0.17888888888888868 |
| Average OMM | AA type | ALA | PLC | 0.066482189 | 0.5591959845636896 | 0.11888888888888867 |
| CS-OMM | AA type | ALA | PLM | 0.080987122 | -0.296294349 | 0.2733333333333328 |
| CS-OMM | AA type | ALA | PLC | 0.18030117557686146 | 0.5184378850452889 | 0.3477777777777776 |
| Average OMM | AA type | GLY | PLM | 0.28619994284234623 | 0.848698348 | 0.33722222222222115 |
| Average OMM | AA type | GLY | PLC | 0.077369981 | 0.5418909170580697 | 0.14277777777777742 |
| CS-OMM | AA type | GLY | PLM | 0.21561620583316202 | 0.29203097855507343 | 0.7383333333333315 |
| CS-OMM | AA type | GLY | PLC | 0.2534077659039931 | 0.5528896710632589 | 0.4583333333333323 |
| Average OMM | AA type | SER | PLM | 0.1558580451378376 | 0.7363372211236433 | 0.21166666666666636 |
| Average OMM | AA type | SER | PLC | -0.001785414 | -0.03418877 | 0.052222222 |
| CS-OMM | AA type | SER | PLM | 0.2477150358594507 | 0.7010802901682586 | 0.3533333333333324 |
| CS-OMM | AA type | SER | PLC | 0.10139449549468633 | 0.7636405518428264 | 0.1327777777777777 |
| Average OMM | AA type | THR | PLM | 0.031649644 | 0.47873411469287874 | 0.066111111 |
| Average OMM | AA type | THR | PLC | 0.00969753 | 0.49873010607262297 | 0.019444444 |
| CS-OMM | AA type | THR | PLM | 0.067806729 | -0.13885337 | 0.48833333333333223 |
| CS-OMM | AA type | THR | PLC | 0.075129616 | -0.461547131 | 0.16277777777777758 |
| Average OMM | AA type | ASN | PLM | 0.049566137 | 0.4622748527005848 | 0.10722222222222204 |
| Average OMM | AA type | ASN | PLC | 0.039130701254932364 | 0.6521783542488739 | 0.06 |
| CS-OMM | AA type | ASN | PLM | 0.002569796 | 0.036711371 | 0.07 |
| CS-OMM | AA type | ASN | PLC | 0.019516412001411028 | 0.11480242353771211 | 0.16999999999999973 |
| Average OMM | AA type | GLN | PLM | 0.17696577520965504 | 0.6677953781496423 | 0.26499999999999974 |
| Average OMM | AA type | GLN | PLC | 0.071419423 | 0.5638375527311992 | 0.12666666666666646 |
| CS-OMM | AA type | GLN | PLM | 0.010065164786115039 | -0.017454043 | 0.5766666666666659 |
| CS-OMM | AA type | GLN | PLC | 0.1833384578639202 | 0.36465107641442746 | 0.502777778 |
| Average OMM | AA type | ASP | PLM | 0.10370023553677545 | 0.36816651669861156 | 0.2816666666666663 |
| Average OMM | AA type | ASP | PLC | 0.054103192 | 0.46154382186315657 | 0.11722222222222207 |
| CS-OMM | AA type | ASP | PLM | 0.28114523093506383 | 0.4013175382102423 | 0.7005555555555546 |
| CS-OMM | AA type | ASP | PLC | 0.1674168722170051 | 0.4012654726905583 | 0.4172222222222221 |
| Average OMM | AA type | GLU | PLM | 0.25824403404537855 | 0.7043019110328528 | 0.36666666666666553 |
| Average OMM | AA type | GLU | PLC | 0.053814616440906404 | 0.36279516701734704 | 0.14833333333333312 |
| CS-OMM | AA type | GLU | PLM | 0.055371542 | -0.054050312 | 1.0244444444444418 |
| CS-OMM | AA type | GLU | PLC | 0.12810072873163045 | 0.31371607 | 0.4083333333333328 |
| Average OMM | AA type | ARG | PLM | 0.068894461 | 0.7294707639631711 | 0.094444444 |
| Average OMM | AA type | ARG | PLC | 0.000841168 | 0.03605005 | 0.023333333333333334 |
| CS-OMM | AA type | ARG | PLM | 0.009938459 | 0.15421746227190486 | 0.064444444 |
| CS-OMM | AA type | ARG | PLC | 0.000177771 | -0.063997537 | 0.002777778 |
| Average OMM | AA type | LYS | PLM | 0.5664094209989786 | 0.9370744097409595 | 0.604444444 |
| Average OMM | AA type | LYS | PLC | 0.15263701959528686 | 0.646462671 | 0.23611111111111086 |
| CS-OMM | AA type | LYS | PLM | 0.32343727829999513 | -0.177496067 | 1.822222222 |
| CS-OMM | AA type | LYS | PLC | 0.022951700771984974 | -0.024723556 | 0.9283333333333318 |
| Average OMM | AA type | HSD | PLM | 0.050297874 | 0.4893847196264258 | 0.10277777777777759 |
| Average OMM | AA type | HSD | PLC | 0.008864345 | 0.21830104146002746 | 0.040606061 |
| CS-OMM | AA type | HSD | PLM | 0.003710773 | 0.016133797 | 0.22999999999999962 |
| CS-OMM | AA type | HSD | PLC | 0.008237251 | 0.080146225 | 0.10277777777777762 |
| Average OMM | AA type | HSE | PLM | -3.87E-05 | -0.06971365 | 0.000555556 |
| Average OMM | AA type | HSE | PLC | 0 |  | 0 |
| CS-OMM | AA type | HSE | PLM | 0.000330173 | -0.084901539 | 0.003888889 |
| CS-OMM | AA type | HSE | PLC | 0.00018424 | -0.331632579 | 0.000555556 |
| Average OMM | AA type | PHE | PLM | 0.058868541400068336 | 0.8278388634384616 | 0.071111111 |
| Average OMM | AA type | PHE | PLC | 0.005201068 | 0.3731200919651253 | 0.013939393939393935 |
| CS-OMM | AA type | PHE | PLM | 0.017024836254163282 | -0.079185285 | 0.2149999999999991 |
| CS-OMM | AA type | PHE | PLC | 0.009847791 | -0.167226641 | 0.058888888888888824 |
| Average OMM | AA type | TYR | PLM | 0.003184312 | 0.6368623140484769 | 0.005 |
| Average OMM | AA type | TYR | PLC | -0.000202478 | -0.111362995 | 0.001818182 |
| CS-OMM | AA type | TYR | PLM | 0.004637654 | -0.050287818 | 0.092222222 |
| CS-OMM | AA type | TYR | PLC | 0 |  | 0 |
| Average OMM | AA type | TRP | PLM | -0.002791803 | -0.173284303 | 0.016111111111111024 |
| Average OMM | AA type | TRP | PLC | 0.001407064 | 0.4643312468173611 | 0.003030303 |
| CS-OMM | AA type | TRP | PLM | 0.091340029 | 0.8792088397173181 | 0.10388888888888881 |
| CS-OMM | AA type | TRP | PLC | 0.004296197 | -0.309326207 | 0.013888888888888833 |
| Average OMM | AA type | PRO | PLM | 0.025289933242060088 | 0.6235873950097017 | 0.040555556 |
| Average OMM | AA type | PRO | PLC | -0.00028105 | -0.231866457 | 0.001212121 |
| CS-OMM | AA type | PRO | PLM | 0.00519559 | -0.027833518 | 0.18666666666666595 |
| CS-OMM | AA type | PRO | PLC | 0.005348482 | 0.2831549431467362 | 0.018888888888888834 |
| Average OMM | AA type | CYS | PLM | -0.001768003 | -0.144654764 | 0.012222222222222218 |
| Average OMM | AA type | CYS | PLC | 0.006456291 | 0.5326440280438489 | 0.012121212121212118 |
| CS-OMM | AA type | CYS | PLM | 0.00789672 | -0.111922018 | 0.070555556 |
| CS-OMM | AA type | CYS | PLC | 0.014591060817932688 | -0.410373586 | 0.035555555555555465 |
| Average OMM | AA type | MET | PLM | 0 |  | 0 |
| Average OMM | AA type | MET | PLC | 0 |  | 0 |
| CS-OMM | AA type | MET | PLM | 8.628009720923702e-05 | 0.1553041749766268 | 0.000555556 |
| CS-OMM | AA type | MET | PLC | 0 |  | 0 |
| Average OMM | Lipid species | DLCL | PLM | -0.000530549 | -0.119373486 | 0.004444444 |
| Average OMM | Lipid species | DLCL | PLC | 0 |  | 0 |
| CS-OMM | Lipid species | DLCL | PLM | 0.544240002 | 0.7192599140767525 | 0.7566666666666647 |
| CS-OMM | Lipid species | DLCL | PLC | 0.14272324021809607 | -0.386318545 | 0.369444444 |
| Average OMM | Lipid species | PLPC | PLM | 0.057923450073417225 | 0.5545862241071865 | 0.10444444444444438 |
| Average OMM | Lipid species | PLPC | PLC | 0.019010548944074633 | 0.26139504798102675 | 0.072727273 |
| CS-OMM | Lipid species | PLPC | PLM | 0.050106175143115717 | -0.205915788 | 0.24333333333333307 |
| CS-OMM | Lipid species | PLPC | PLC | 0.084094635 | -0.24180566 | 0.3477777777777769 |
| Average OMM | Lipid species | PLPE | PLM | 0.042088411422799996 | 0.6764208978664294 | 0.062222222 |
| Average OMM | Lipid species | PLPE | PLC | 0.012139879 | 0.3641963770964634 | 0.033333333 |
| CS-OMM | Lipid species | PLPE | PLM | 0.010557478599626791 | -0.109215296 | 0.096666667 |
| CS-OMM | Lipid species | PLPE | PLC | 0.10814429400260712 | 0.9358640827148705 | 0.11555555555555541 |
| Average OMM | Lipid species | POCL | PLM | 0 |  | 0 |
| Average OMM | Lipid species | POCL | PLC | 0 |  | 0 |
| CS-OMM | Lipid species | POCL | PLM | 0.021915646453860804 | 0.17689759469484065 | 0.12388888888888884 |
| CS-OMM | Lipid species | POCL | PLC | 0.020082195064851215 | -0.331632579 | 0.060555556 |
| Average OMM | Lipid species | POPC | PLM | 0.010744890219463047 | 0.064902021 | 0.16555555555555543 |
| Average OMM | Lipid species | POPC | PLC | 0.033340069 | 0.5613378909389778 | 0.059393939 |
| CS-OMM | Lipid species | POPC | PLM | 0.037862222 | -0.082110844 | 0.46111111111111075 |
| CS-OMM | Lipid species | POPC | PLC | 0.009260455 | -0.106851407 | 0.086666667 |
| Average OMM | Lipid species | POPE | PLM | 0.085249362 | 0.7071375677450779 | 0.12055555555555542 |
| Average OMM | Lipid species | POPE | PLC | 0.006300324 | 0.1856345425234818 | 0.033939394 |
| CS-OMM | Lipid species | POPE | PLM | 0.031731735929884784 | -0.06965503 | 0.4555555555555542 |
| CS-OMM | Lipid species | POPE | PLC | 0.14823659395385116 | 0.6755085294099569 | 0.21944444444444375 |
| Average OMM | Lipid species | POPI | PLM | -0.00193003 | -0.157911564 | 0.012222222222222167 |
| Average OMM | Lipid species | POPI | PLC | 0.042360005 | 0.5460469364451267 | 0.077575758 |
| CS-OMM | Lipid species | POPI | PLM | 0 |  | 0 |
| CS-OMM | Lipid species | POPI | PLC | 0 |  | 0 |
| Average OMM | Lipid species | SAPC | PLM | 0.39303982339116833 | 0.8188329653982704 | 0.4799999999999982 |
| Average OMM | Lipid species | SAPC | PLC | 0.1419597709084383 | 0.5263676898852213 | 0.2696969696969694 |
| CS-OMM | Lipid species | SAPC | PLM | 0.011338569949788683 | 0.015994848 | 0.7088888888888872 |
| CS-OMM | Lipid species | SAPC | PLC | 0.15577413148789257 | -0.331826552 | 0.4694444444444437 |
| Average OMM | Lipid species | SAPE | PLM | 0.3105124626144151 | 0.7315738647983612 | 0.4244444444444439 |
| Average OMM | Lipid species | SAPE | PLC | -0.015481717 | -0.220214075 | 0.07030303 |
| CS-OMM | Lipid species | SAPE | PLM | 0.1496940532417338 | 0.11292929414715903 | 1.3255555555555525 |
| CS-OMM | Lipid species | SAPE | PLC | 0.13868212562718907 | 0.2752236230749072 | 0.5038888888888878 |
| Average OMM | Lipid species | SAPI | PLM | 0.10894041495339461 | 0.7571148529579556 | 0.14388888888888876 |
| Average OMM | Lipid species | SAPI | PLC | 0.003207583 | 0.14701423203960212 | 0.021818181818181778 |
| CS-OMM | Lipid species | SAPI | PLM | 0.002637205 | 0.010341981240537094 | 0.25499999999999984 |
| CS-OMM | Lipid species | SAPI | PLC | 0.073843676 | 0.5381320503354554 | 0.13722222222222208 |
| Average OMM | Lipid species | SAPS | PLM | 0.076302821 | 0.7116325295134223 | 0.10722222222222215 |
| Average OMM | Lipid species | SAPS | PLC | 0.00175492 | 0.3619523491310562 | 0.004848485 |
| CS-OMM | Lipid species | SAPS | PLM | 0.003522124 | -0.045940745 | 0.076666667 |
| CS-OMM | Lipid species | SAPS | PLC | 0.037126892 | 0.6488194733117563 | 0.057222222 |
| Average OMM | Lipid species | SEPI | PLM | 0.007728196 | 0.6624167968706918 | 0.011666666666666667 |
| Average OMM | Lipid species | SEPI | PLC | 0.007019682 | 0.36195234913105623 | 0.019393939393939363 |
| CS-OMM | Lipid species | SEPI | PLM | 0.014562559746474886 | -0.081659214 | 0.17833333333333323 |
| CS-OMM | Lipid species | SEPI | PLC | 0.057040175 | 0.34569803213298106 | 0.16499999999999979 |
| Average OMM | Lipid species | SLCL | PLM | 0 |  | 0 |
| Average OMM | Lipid species | SLCL | PLC | 0 |  | 0 |
| CS-OMM | Lipid species | SLCL | PLM | 0 |  | 0 |
| CS-OMM | Lipid species | SLCL | PLC | 0.013449543483799425 | -0.331632579 | 0.040555555555555504 |
| Average OMM | Lipid species | SLPA | PLM | -0.000770589 | -0.198151357 | 0.003888889 |
| Average OMM | Lipid species | SLPA | PLC | -6.75E-05 | -0.111362995 | 0.000606061 |
| CS-OMM | Lipid species | SLPA | PLM | 0.021714251444638753 | 0.7976663795989765 | 0.027222222222222158 |
| CS-OMM | Lipid species | SLPA | PLC | 0 |  | 0 |
| Average OMM | Lipid species | SLPC | PLM | 0.10490401972886779 | 0.6488908436837194 | 0.16166666666666638 |
| Average OMM | Lipid species | SLPC | PLC | 0.059111429 | 0.5510387456343521 | 0.10727272727272714 |
| CS-OMM | Lipid species | SLPC | PLM | 0.10618980412105089 | 0.2896085566937757 | 0.366666667 |
| CS-OMM | Lipid species | SLPC | PLC | 0.041567641 | -0.448034455 | 0.092777778 |
| Average OMM | Lipid species | SLPE | PLM | 0.18746326812972683 | 0.7651561964478656 | 0.2449999999999997 |
| Average OMM | Lipid species | SLPE | PLC | 0.012929626171168485 | 0.19219714578763997 | 0.067272727 |
| CS-OMM | Lipid species | SLPE | PLM | 0.058136032744861905 | -0.154800087 | 0.37555555555555525 |
| CS-OMM | Lipid species | SLPE | PLC | 0.044124349 | 0.1644385667761983 | 0.26833333333333237 |
| Average OMM | Lipid species | SLPI | PLM | 0.010386021414480999 | 0.096365147 | 0.1077777777777776 |
| Average OMM | Lipid species | SLPI | PLC | 0.014524303454569593 | 0.15461355290348297 | 0.093939394 |
| CS-OMM | Lipid species | SLPI | PLM | 0.075818358 | 0.5933610657483864 | 0.12777777777777777 |
| CS-OMM | Lipid species | SLPI | PLC | 0.004435349 | -0.27529752 | 0.016111111111111073 |
| Average OMM | Lipid species | SOCL | PLM | 0 |  | 0 |
| Average OMM | Lipid species | SOCL | PLC | 0 |  | 0 |
| CS-OMM | Lipid species | SOCL | PLM | 0.014832497 | -0.125936296 | 0.11777777777777722 |
| CS-OMM | Lipid species | SOCL | PLC | 0.038568713 | 0.6488194733117564 | 0.059444444 |
| Average OMM | Lipid species | SOPI | PLM | 0.020931566 | 0.49574760418284886 | 0.042222222 |
| Average OMM | Lipid species | SOPI | PLC | 0.022783081154079392 | 0.3417462173111913 | 0.066666667 |
| CS-OMM | Lipid species | SOPI | PLM | 0.025486245 | -0.086394051 | 0.2949999999999999 |
| CS-OMM | Lipid species | SOPI | PLC | 0.078994248 | 0.7769926054788128 | 0.10166666666666657 |
| Average OMM | Lipid type | CL | PLM | -0.000530549 | -0.119373486 | 0.004444444 |
| Average OMM | Lipid type | CL | PLC | 0 |  | 0 |
| CS-OMM | Lipid type | CL | PLM | 0.5773014350150595 | 0.5782652103656704 | 0.998333333 |
| CS-OMM | Lipid type | CL | PLC | 0.10710086694503423 | -0.202077107 | 0.5299999999999995 |
| Average OMM | Lipid type | PC | PLM | 0.6790211130169036 | 0.7448129210423092 | 0.9116666666666644 |
| Average OMM | Lipid type | PC | PLC | 0.2892969738517389 | 0.568261913 | 0.5090909090909078 |
| CS-OMM | Lipid type | PC | PLM | 0.12295048417256839 | 0.069073306 | 1.779999999999995 |
| CS-OMM | Lipid type | PC | PLC | 0.357565646 | -0.358761517 | 0.9966666666666638 |
| Average OMM | Lipid type | PE | PLM | 0.6743274681983679 | 0.7912577853696641 | 0.8522222222222204 |
| Average OMM | Lipid type | PE | PLC | 0.043089382 | 0.2103475770999709 | 0.20484848484848459 |
| CS-OMM | Lipid type | PE | PLM | 0.011187081432374705 | -0.004964681 | 2.253333333 |
| CS-OMM | Lipid type | PE | PLC | 0.5601889082565115 | 0.5059408102667948 | 1.1072222222222206 |
| Average OMM | Lipid type | PS | PLM | 0.076302821 | 0.7116325295134223 | 0.10722222222222215 |
| Average OMM | Lipid type | PS | PLC | 0.00175492 | 0.3619523491310562 | 0.004848485 |
| CS-OMM | Lipid type | PS | PLM | 0.003522124 | -0.045940745 | 0.076666667 |
| CS-OMM | Lipid type | PS | PLC | 0.037126892 | 0.6488194733117563 | 0.057222222 |
| Average OMM | Lipid type | PA | PLM | -0.000770589 | -0.198151357 | 0.003888889 |
| Average OMM | Lipid type | PA | PLC | -6.75E-05 | -0.111362995 | 0.000606061 |
| CS-OMM | Lipid type | PA | PLM | 0.021714251444638753 | 0.7976663795989765 | 0.027222222222222158 |
| CS-OMM | Lipid type | PA | PLC | 0 |  | 0 |
| Average OMM | Lipid type | PI | PLM | 0.21140110750300864 | 0.6652482403940847 | 0.31777777777777705 |
| Average OMM | Lipid type | PI | PLC | 0.13694822880792865 | 0.49016177339063477 | 0.2793939393939389 |
| CS-OMM | Lipid type | PI | PLM | 0.11317178323955152 | 0.13219286815781506 | 0.8561111111111099 |
| CS-OMM | Lipid type | PI | PLC | 0.2557656014737726 | 0.608965718 | 0.4199999999999997 |
