# Supplementary material for: Long-chain fatty acid- and acylcarnitine-bound oxygenated myoglobin interactions with outer mitochondrial membrane
Source: Front Bioinform. 2026 Oct 16;6:1986755. doi: 10.3389/fbinf.2026.1986755 (PMC13719540; doi:10.3389/fbinf.2026.1986755)
Supplement: Supplementary file 3 [file Table3.docx]

**Supplementary Table 3.** Contribution of the amino acid and lipid types to the stability of mouse Mb-mitochondrial membrane contact in steered detachment simulations for the (A) average OMM and (B) contact-sites OMM. The average contact frequency was estimated as time-averaged number of contacts for heavy atoms for the residue of a particular type. The contact frequency in the table is color-coded from white (the lowest number of contacts) to green (the highest). Correlation of particular residue type contacts with the average force required for detachment was calculated based on the whole set of simulations for the given system type (e.g., deoxy-Mb with the contact sites of OMM), up to 12 independent simulated detachments as described in Methods. Correlation cells are color-coded from red (negative correlation) to blue (positive). The importance score is a product of the correlation with the energy and the average frequency of contacts and color-coded from white to burnt orange.
