# Supplementary material for: Long-chain fatty acid- and acylcarnitine-bound oxygenated myoglobin interactions with outer mitochondrial membrane
Source: Front Bioinform. 2026 Oct 16;6:1986755. doi: 10.3389/fbinf.2026.1986755 (PMC13719540; doi:10.3389/fbinf.2026.1986755)
Supplement: Supplementary file 4 [file Table4.docx]

**Supplementary Table 4**. **Previously reported steered-detachment forces for oxy-Mb interacting with the average OMM and CS-OMM.** Average forces required to detach oxy-Mb from the average-composition outer mitochondrial membrane (OMM) and cardiolipin-enriched contact-site OMM (CS-OMM) are shown as a reference for comparison with the PLM- and PLC-bound oxy-Mb systems examined in the present study. Detachment forces were obtained from 30-ns steered molecular dynamics simulations using constant-velocity movement of the harmonically restrained protein center of mass. Individual cells represent independent Mb–membrane configurations; empty cells indicate configurations that did not retain sufficient membrane contact for steered-detachment analysis. The average detachment force was 32.4 pN for oxy-Mb at the average OMM and 66.7 pN at the CS-OMM, corresponding to a CS-OMM/average-OMM ratio of 2.1. *Data were previously reported in Anishkin et al. (2023) and are reproduced here for direct comparison with the lipid-bound oxy-Mb systems analyzed in the present study.*
